# Two‐Pronged Attack: Dual Activation of Fat Reduction Using Near‐Infrared‐Responsive Nanosandwich for Targeted Anti‐Obesity Treatment

**DOI:** 10.1002/advs.202406985

**Published:** 2024-09-26

**Authors:** Qiaqia Xiao, Lu Tang, Siying Chen, Yijun Mei, Chuying Wang, Jing Yang, Jing Shang, Shengliang Li, Wei Wang

**Affiliations:** ^1^ State Key Laboratory of Natural Medicines School of Pharmacy China Pharmaceutical University Nanjing 211198 P. R. China; ^2^ NMPA Key Laboratory for Research and Evaluation of Cosmetics China Pharmaceutical University Nanjing 211198 P. R. China; ^3^ School of Traditional Chinese Pharmacy China Pharmaceutical University Nanjing 211198 P. R. China; ^4^ College of Pharmaceutical Sciences Soochow University Suzhou 215123 P. R. China

**Keywords:** anti‐inflammation, black phosphorus nanosheet, lipid reduction, nanomedicine, obesity

## Abstract

Excessive fat accumulation and chronic inflammation are two typical characteristics of obesity. AMP‐activated protein kinase (AMPK), a master regulator of energy metabolism, is involved in adipogenesis, lipogenesis, and inflammation modulation in adipose tissue (AT). Thus, effective lipid reduction and anti‐inflammation through AMPK regulation play vital roles in treating obesity. Herein, an anti‐obesity nanosandwich is fabricated through attaching polymetformin (PolyMet) onto photothermal agent black phosphorus nanosheets (BP). PolyMet activates AMPK to inhibit adipogenesis, promote browning, and mitigate AT inflammation by decreasing macrophage infiltration, repolarizing macrophage phenotype, and downregulating pro‐inflammatory cytokines. Additionally, BP induces lipolysis and apoptosis of adipocytes and macrophages through a photothermal effect. By further functionalization using hyaluronic acid (HA) and MMP2 substrate‐linking P3 peptide‐modified HA (P3‐HA), an enhanced anti‐obesity effect is obtained by dual‐targeting of P3 and HA, and HA‐mediated CD44 poly‐clustering after MMP2 cleavage. Upon laser irradiation, the designed nanosandwich (P3‐HA/PM@BP) effectively inhibits obesity development in obese mice, increases M2/M1 ratio in AT, reduces the serum levels of cholesterol/triglyceride and improves insulin sensitivity, exhibiting promising research potential to facilitate the clinical development of modern anti‐obesity therapies.

## Graphical abstract

This work develops an anti‐obesity P3‐HA/PM@BP nanosandwich that targets white adipose tissue and poly‐clusters CD44 receptors on adipocytes and macrophages. Upon near‐infrared laser irradiation, this nanosandwich effectively exerts lipid reduction and anti‐inflammation functions through photothermal therapy and activation of AMPK‐related pathways, eventually leading to a significant weight loss of obese mice and relieving obesity‐associated disorders.

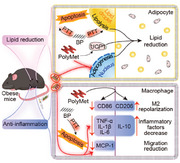

## 1 Introduction

Obesity is an epidemic metabolic disease, in which excess energy is stored as fat and accompanied by low‐grade chronic inflammation, which is regarded as an overwhelming challenge to public health.^[^ ^1^ ^]^ However, in addition to the daily optimization of diet, exercise, or surgery, many currently available small‐molecule drugs that either inhibit the absorption of dietary fat in the gastrointestinal tract such as Orlistat®, or suppress appetite in the central nervous system like Liraglutide®, all suffer from side effects and limited anti‐obesity efficacies due to off‐target effect and long‐term use.^[^ ^2^ ^]^ Therefore, it is urgent to develop safe and efficient alternatives to treat obesity and its associated disorders.

Ablation of adipose tissue (AT) by photothermal therapy (PTT) is a direct way to get rid of excess fat. In some reported studies, the photothermal effect of gold nanoparticles (NPs) and CuS nanodots was utilized to shrink adipocytes.^[^ ^3^ ^]^ Compared to those photothermal agents, black phosphorus nanosheets (BP) with excellent photothermal conversion efficiency hold unique advantages such as good biocompatibility and biodegradability, high surface‐to‐volume ratio because of their wrinkled lattice configuration, and easy modifiability, endowing them with reliable biosafety and satisfactory drug loading capacity.^[^ ^4^ ^]^ Meanwhile, BP can be simply exfoliated in liquid phase to obtain suitable particle size, making them ideal drug delivery vehicles in biomedical fields.^[^ ^5^ ^]^ Based on these merits, BP‐mediated nanoplatforms show great potential in anti‐obesity based on photothermal treatment. However, the intrinsic chronic inflammation in AT may impair the overall therapeutic efficacy against obesity.^[^ ^6^ ^]^ Adipose tissue macrophage (ATM) accounts for 5% of the total cells in normal AT, surprisingly, ATM can elevate to 50% in obese AT due to the influx of bone marrow‐originated precursors and subsequent differentiation to F4/80‐expressing macrophages.^[^ ^7^ ^]^ Moreover, M2‐like macrophages tend to repolarize into M1‐like macrophages due to the abundant free fatty acids (FFA), tumor necrosis factor‐alpha (TNF‐α), and lipopolysaccharide (LPS) in AT.^[^ ^8^ ^]^ Pro‐inflammatory factors secreted by M1 are also able to exacerbate adipocyte fibrosis and systemic metabolic disorder through extracellular matrix (ECM) remodeling triggered by upregulated matrix metalloproteinases 2 (MMP2).^[^ ^9^ ^]^ Therefore, combining lipid reduction with inflammation relief is a feasible strategy for anti‐obesity treatment.

Metformin (Met) is the first‐line oral hypoglycemic drug for the treatment of type 2 diabetes (T2D) and its favorable effect on weight loss has been extensively confirmed.^[^ ^10^ ^]^ Met downregulates the expression of peroxisome proliferators‐activated receptor gamma (PPARγ) and CAAT‐enhancer‐binding protein alpha (C/EBPα) in 3T3‐L1 cells after activating AMP‐activated protein kinase (AMPK), which inhibits adipogenesis and lipogenesis.^[^ ^11^ ^]^ In addition, it has been demonstrated that Met reduces the levels of monocyte chemotactic protein‐1 (MCP‐1), M1 markers such as CD11c, and pro‐inflammatory cytokines such as interleukin‐6 (IL‐6) and TNF‐α in obese AT, indicating the anti‐inflammation effects of Met through the mechanisms associated with decreased macrophage infiltration, M2 macrophage repolarization and declined secretion of inflammatory factors.^[^ ^12^ ^]^ In our previous study, polymetformin (PolyMet), which contained a large number of biguanide groups, was synthesized by the addition reaction of chitosan and dicyandiamide.^[^ ^13^ ^]^ In this present work, it is expected that PolyMet would serve dual functions in lipid reduction and anti‐inflammation against obesity as Met. Hyaluronic acid (HA) is a linear glycosaminoglycan that participates in cell proliferation and metastasis through specifically binding to CD44 receptors.^[^ ^14^ ^]^ It has been demonstrated that the molecular weight and dispersion state of HA affects its roles on cells, and HA with high molecular weight (HMW) or HA‐shelled NPs could target and poly‐cluster CD44 receptors on the surface of adipocytes or macrophages, which in turn blocked the downstream signaling pathways and ultimately inhibited adipogenesis and inflammation in obese mice.^[^ ^15^ ^]^ In addition, the short P3 peptide (CKGGRAKDC) could specifically bind to prohibitin (PHB), which is highly expressed on the adipose tissue vasculature and the membranes of mature adipocytes, thereby enhancing the targeting of AT.^[^ ^16^ ^]^

The electrostatic layer‐by‐layer (LbL) technique for the fabrication of multi‐layered nanostructures has attracted the interest of researchers due to its versatility and relatively simple procedures.^[^ ^17^ ^]^ Fabrication of multi‐layered nanostructures using the LbL method involves several distinct stages including a selection of a template and a pair of polyelectrolytes with opposite charges and adsorption of two polyelectrolytes onto the selected template to form multi‐layered nanostructures due to the existence of electrostatic adsorption and other interactions between the two polyelectrolytes.^[^ ^18^ ^]^ The layer number of multi‐layer nanostructures is determined by the intrinsic properties of selected materials and the external preparation conditions such as temperature, pH, and ionic strength.^[^ ^19^ ^]^ It is demonstrated that BP is reactive to oxygen and water to form phosphate ions on the surface, rendering it electronegative property.^[^ ^20^ ^]^ PolyMet was prepared from acidified chitosan that carries abundant positive charges in an aqueous solution due to the protonation of amino groups.^[^ ^13a^ ^]^ Therefore, positively charged PolyMet could be attached on negatively charged BP by electrostatic adsorption, eventually forming a positively charged PM@BP nanocomposite. By virtue of a large number of carboxyl groups, HA and MMP2 substrate‐linking P3 peptide‐modified HA (P3‐HA) are negatively charged, which can be subsequently adsorbed onto positively charged PM@BP through electrostatic interaction to construct the final P3‐HA/PM@BP nanosandwich.

**Scheme 1:**
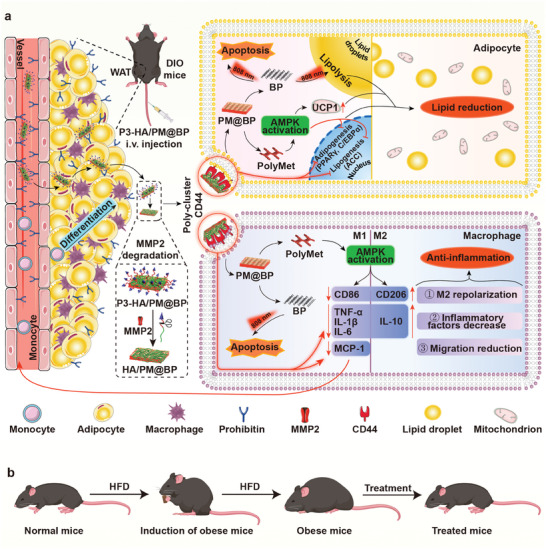
Schematic illustration of obesity treatment by P3‐HA/PM@BP nanosandwich. a) Mechanisms of proposed anti‐obesity treatment based on the “lipid reduction and anti‐inflammation” strategy generated by P3‐HA/PM@BP nanosandwich. b) The treatment circle of obese mice.

Based on this, a “lipid reduction and anti‐inflammation” strategy was proposed by fabricating P3‐HA/PM@BP nanosandwich for anti‐obesity treatment. In this nanosandwich, PolyMet, HA, and P3‐HA were self‐assembled in an LbL manner to deposit on BP via electrostatic interaction. After intravenous injection, P3‐HA/PM@BP nanosandwich targeted adipocytes and macrophages in obese AT dependent on the dual guidance of P3 and HA, which subsequently poly‐clustered CD44 with exposed HA after MMP2 degradation to inhibit lipogenesis and inflammation. Simultaneously, these inhibitory effects on lipogenesis and inflammation were further facilitated by PolyMet through AMPK activation. Upon laser irradiation, BP‐triggered photothermal effect induced apoptosis and lipolysis, leading to the shrinkage of adipocytes in AT (**Scheme** **1**a). As a result, P3‐HA/PM@BP nanosandwich plus laser treatment improved the whole metabolism including reduced body weight, decreased serum lipid profiles, and increased insulin sensitivity (Scheme 1b). Collectively, the designed strategy in this work demonstrated a fresh opportunity for obesity management, which provided reasonable research implications in precise body weight control, exhibiting the potential to be applied in preventing obesity‐associated metabolic disorders.

## 2 Results and Discussion

### 2.1 Preparation and Characterization of PolyMet

**Figure 1:**
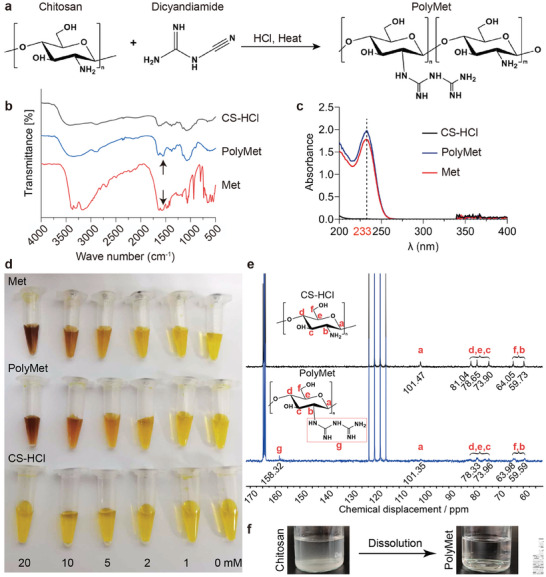
Preparation and characterization of PolyMet. a) Synthesis route of PolyMet. b) FT‐IR spectra, c) UV–vis spectra (range of 200–400 nm), and d) color test of PolyMet, CS‐HCl, and Met. e) ^13^C NMR spectra of PolyMet and CS‐HCl. f) Water solubility of PolyMet compared to chitosan.

PolyMet, which contains a large number of biguanide groups, was synthesized using the previously published methods by the reaction of hydrochloride chitosan (CS‐HCl) and dicyandiamide under high temperature and acidic conditions as the synthesis route shown in **Figure** **1**a.^[^ ^13a^ ^]^ Fourier transform infrared spectra (FT‐IR) were employed to verify the structure of PolyMet, which revealed that PolyMet possessed the absorption peak of biguanide groups at ≈1600 cm^−1^ like Met, indicating the successful introduction of biguanide groups into the chitosan chain as previously published (Figure 1b).^[^ ^21^ ^]^ In addition, the successful synthesis of PolyMet was further verified by its UV–vis spectra and color test by mixing it with the solution of sodium nitroprusside/potassium hexacyanoferrate (III)/NaOH. As shown in Figure 1c, compared to CS‐HCl, both PolyMet and Met exhibited a characteristic peak at 233 nm. Besides, after mixing with the chromogenic agent for 10 min in the dark, the color of both PolyMet and Met solutions changed from yellow to red, and the chromogenic effect became more pronounced when the concentration of the biguanide group increased. By contrast, the CS‐HCl solution remained yellow as the original color of the chromogenic agent despite of its increased concentration (Figure 1d). Moreover, ^13^C NMR spectroscopy was performed to validate the successful synthesis of PolyMet as well (Figure 1e). It was shown that CS‐HCl and PolyMet shared the same absorption peaks at 164.84, 164.52, 164.20, 163.88, 122.24, 119.97, 117.69, and 115.42 ppm, which corresponded to the peaks of deuterium reagent deuterotrifluoroacetic acid. In addition, both CS‐HCl and PolyMet spectra contained characteristic peaks of chitosan monomers at C1 (a), C4 (d), C5 (e), C3 (c), C6 (f), and C2 (b), and there were characteristic biguanide groups in PolyMet at the peak of 158.32 ppm (g), thus indicating that PolyMet was successfully prepared. In addition, the solubility of CS after introducing biguanide groups improved significantly in aqueous conditions with neutral pH, making PolyMet beneficial for biological applications (Figure 1f).

### 2.2 Preparation and Characterization of P3‐HA/PM@BP Nanosandwich

**Figure 2:**
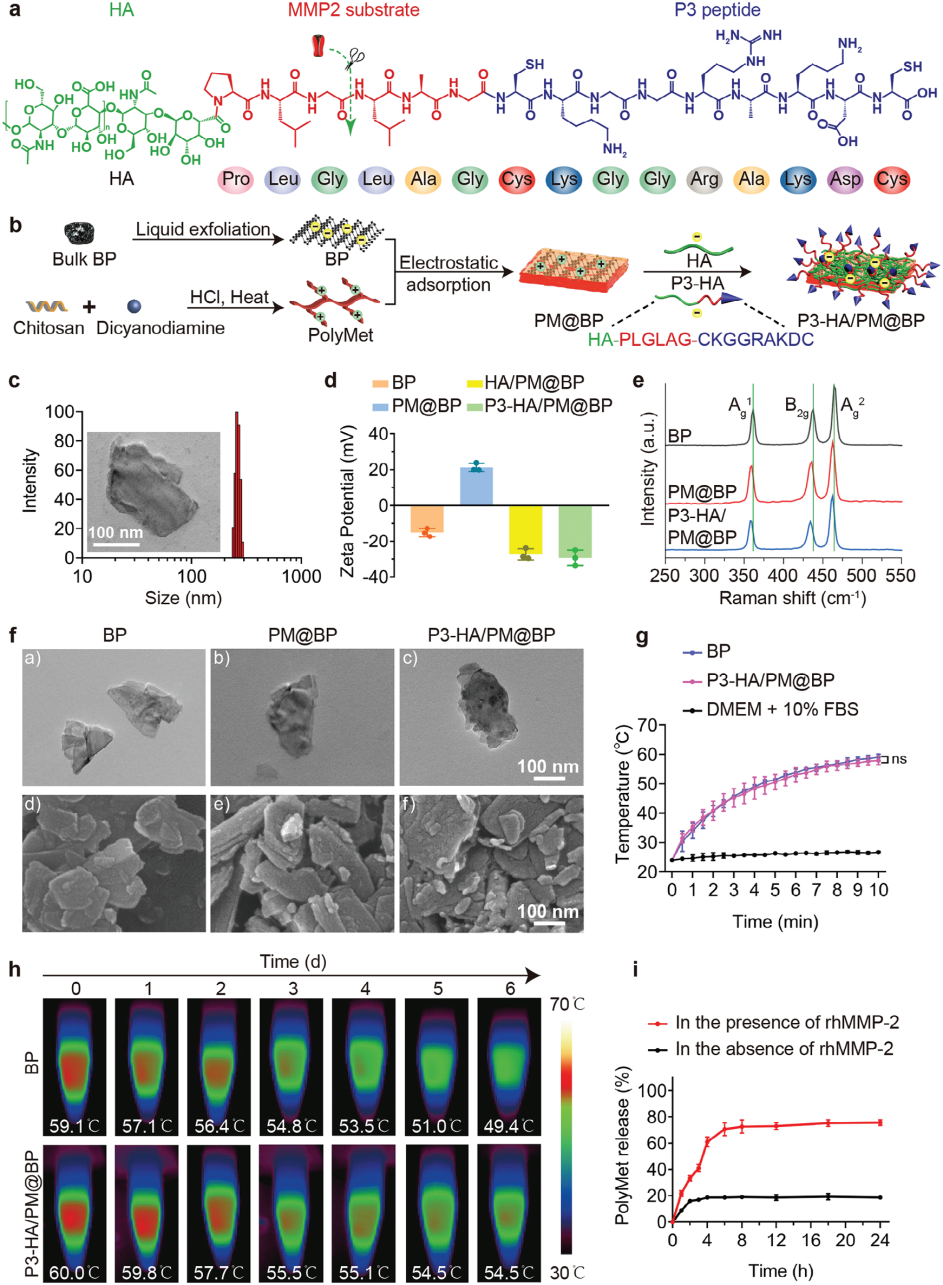
Preparation and characterization of P3‐HA/PM@BP nanosandwich. a) Schematic illustration of the composition of P3‐HA. b) Schematic illustration of the construction process of P3‐HA/PM@BP nanosandwich. c) Hydrodynamic diameters and inserted TEM image of P3‐HA/PM@BP. Scale bar = 100 nm. d) Zeta potentials of BP, PM@BP, HA/PM@BP, and P3‐HA/PM@BP (*n* = 3). e) Raman spectra, f) TEM a‐c) and SEM d‐f) of BP, PM@BP, and P3‐HA/PM@BP. g) Temperature profiles of BP (100 µg mL^−1^) and P3‐HA/PM@BP in DMEM containing 10% FBS upon NIR laser irradiation (808 nm, 1.5 W cm^−2^) (*n* = 3), and h) the NIR thermographs within one week. i) Drug release profiles of P3‐HA/PM@BP in the presence or absence of rhMMP2 enzyme (10 nM) (*n* = 3). One‐way ANOVA followed by Tukey's multiple comparisons test was used for comparisons among multiple groups: ns indicated *P* > 0.05.

Next, P3‐HA/PM@BP nanosandwich was self‐assembled in layer‐by‐layer (LbL) manner through BP, PolyMet, HA, and MMP2 substrate‐linking P3 peptide‐modified HA (P3‐HA, HA‐PLGLAG‐CKGGRAKDC) as depicted in **Figure** **2**a,b. In brief, P3‐HA/PM@BP was mainly obtained by LbL deposition of positively charged PolyMet, negatively charged HA, and P3‐HA into the negatively charged BP through electrostatic interaction. Accompanied by the adsorptions of PolyMet, HA, and P3‐HA, the hydrodynamic diameters of fabricated nanosandwich increased gradually from 173.5 ± 5.2 nm to 258.6 ± 5.9 nm (Figure 2c; Figure S1, Supporting Information). Meanwhile, the zeta potentials reversed twice from ‐15.13 ± 1.31 mV to 21.21 ± 1.32 mV, and finally to ‐29.36 ± 2.47 mV in P3‐HA/PM@BP nanosandwich (Figure 2d). The changes of particle sizes and zeta potentials during the preparation process confirmed the successful construction of P3‐HA/PM@BP nanosandwich. In addition, as the Raman spectra in Figure 2e implied, all samples showed distinct characteristic peaks at ≈361.388, 437.719, and 464.192 cm^−1^, which corresponded to A_g_^1^, B_2g_, and A_g_^2^ modes of BP, respectively. Compared to the characteristic peaks of BP, the peaks of PM@BP slightly shifted to lower wavenumbers, while the peaks of P3‐HA/PM@BP nanosandwich shifted more. These changes in the peak positions due to the variations in nanocomplexes (NCs) thickness further supported the successful construction of P3‐HA/PM@BP. Besides, the stepwise surface modifications of BP were visualized by morphology alterations observed under transmission electron microscopy (TEM) and scanning electron microscopy (SEM) (Figure 2f). BP was characterized by a thin layer with a flat surface, and PM@BP was featured by a smooth surface with increased thickness, while the surface of P3‐HA/PM@BP was rough and spotted. In addition, fluorescence co‐localization was performed to prove the successful preparation of P3‐HA/PM@BP. The green fluorescence of FITC‐labeled PolyMet (FITC‐PM) overlapped with the red fluorescence of Cy3‐labeled P3‐HA (Cy3‐P3), emitting distinct yellow fluorescence from (Cy3‐P3)‐HA/(FITC‐PM)@BP. Moreover, the red fluorescence from Cy3‐P3 was wrapped around the yellow fluorescence, indicating that P3‐HA is attached to the surface of the nanosandwich (Figure S2, Supporting Information). Furthermore, by measuring the contents of free PolyMet in the supernatant after ultrafiltration during the preparation process, the drug loading efficiency (DLE) of PolyMet was calculated to be 25.47 ± 1.68%.

Because P3‐HA/PM@BP were mainly constructed via electrostatic adsorption that tends to get collapse due to charged proteins such as serum albumin and lipoprotein in blood, the stability of P3‐HA/PM@BP was tested in different conditions.^[^ ^22^ ^]^ The hydrodynamic diameter maintained stable in different media including pure water, PBS (pH 7.4), and DMEM containing 10% FBS within one week, indicating that the therapeutic efficacy of P3‐HA/PM@BP would not be affected during blood circulation (Figure S3, Supporting Information). Besides, the photothermal properties of P3‐HA/PM@BP are displayed in Figure 2g. Upon near‐infrared (NIR) laser irradiation (808 nm, 1.5 W cm^−2^), the temperature of P3‐HA/PM@BP suspended in DMEM containing 10% FBS increased from 24.0 ± 0.1 °C to 57.9 ± 0.8 °C within 10 min, which showed nearly the same PTT efficacy as free BP, while negligible temperature increase was observed in the medium group. Compared to bare BP, surface functionalization prevented its degradation in the medium, which was demonstrated by the relatively stable photothermal effect of P3‐HA/PM@BP within one week (Figure 2h). Moreover, the photothermal stability of P3‐HA/PM@BP was further verified by the unaffected temperature profiles after 5 repeated laser irradiation cycles (Figure S4, Supporting Information). In conclusion, all the investigations verified that compared to bare BP, surface dense modification with PolyMet and HA not only maintained the photothermal effect of BP but also prevented the oxidation and degradation of BP nanocarrier in physiological conditions, enabling the practical applications of P3‐HA/PM@BP in vivo.

Moreover, the release profiles of PolyMet from P3‐HA/PM@BP nanosandwich were evaluated under both normal and pathological AT‐mimicking conditions. As demonstrated in Figure 2i, only 18.67 ± 0.88% of PolyMet released in PBS during 24 h, while 70.67 ± 2.91% of them released within 6 h in the presence of recombinant human MMP2 (rhMMP2), indicating that MMP2 was conducive to collapse P3‐HA/PM@BP to facilitate the release of PolyMet, further verifying the good responsiveness of P3‐HA/PM@BP nanosandwich to MMP2 enzyme.

### 2.3 Cellular Uptake and CD44 Clustering In Vitro

**Figure 3:**
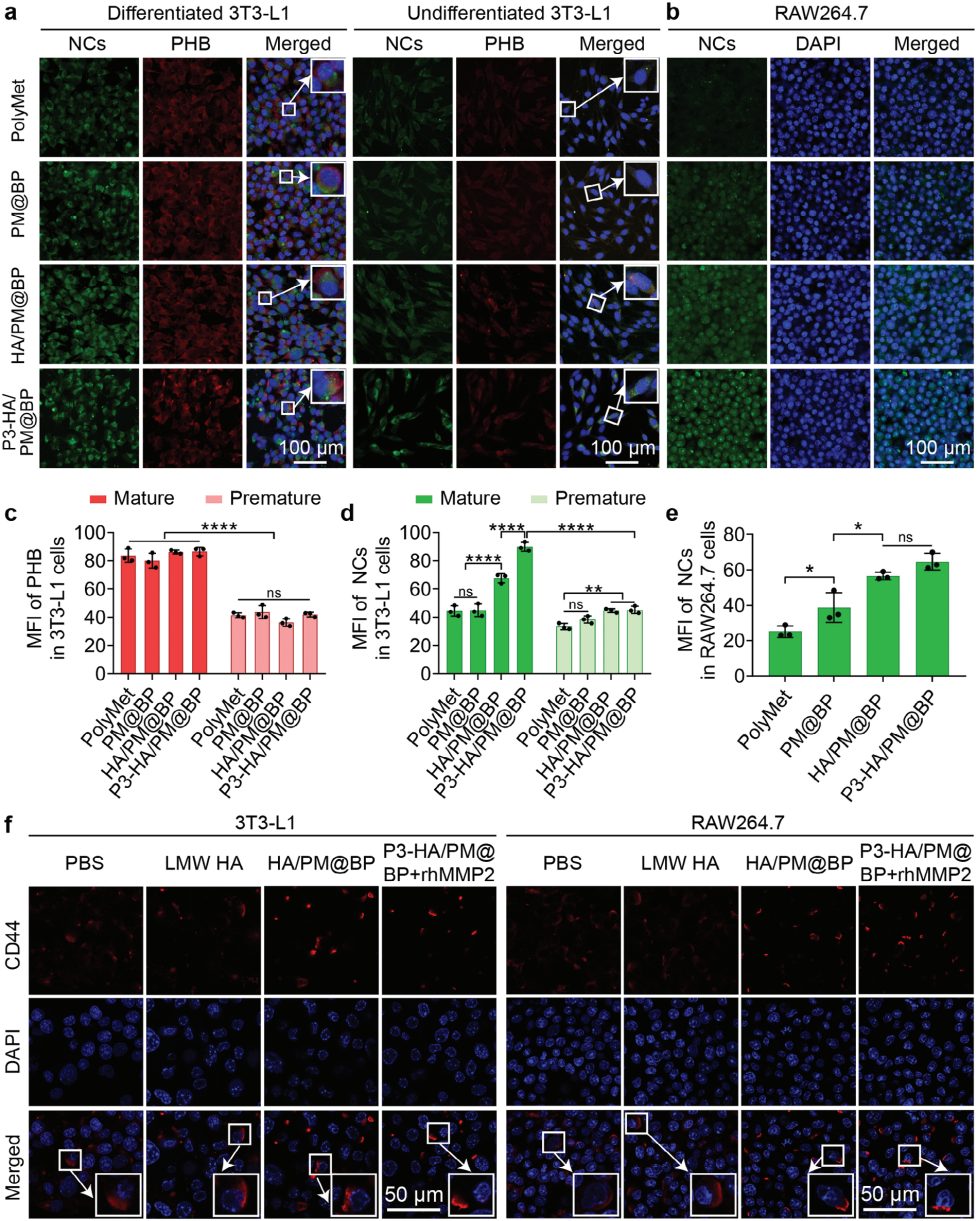
Cellular uptake and CD44 clustering of P3‐HA/PM@BP nanosandwich in vitro. a) CLSM images of NCs and PHB in differentiated and undifferentiated 3T3‐L1 cells, and b) CLSM images of NCs in RAW264.7 cells after incubation with PolyMet, PM@BP, HA/PM@BP, and P3‐HA/PM@BP respectively. The quantified MFI of c) PHB, and NCs in d) 3T3‐L1 cells and e) RAW264.7 cells (*n* = 3). PHB were immune‐stained with Cy3 (red), NCs were labeled with FITC (green), and the nuclei were stained with DAPI (blue). f) The immunofluorescent staining of CD44 poly‐clustering in 3T3‐L1 and RAW264.7 cells. The CD44 receptors on the cell membrane were stained with Cy3 (red), and the nuclei were stained with DAPI (blue). One‐way ANOVA followed by Tukey's multiple comparisons test was used for comparisons among multiple groups: ns indicated *p* > 0.05, ^*^ *p* < 0.05, ^**^ *p* < 0.01, ^****^ *p* < 0.0001.

PHB is an evolutionarily conserved and ubiquitously expressed protein that functions in mitochondrial functional homeostasis and cell cycle regulation. It is expressed primarily in the inner mitochondrial membrane and cytoplasm of preadipocytes but translocated to the cell membrane and nucleus of mature adipocytes. Meanwhile, PHB is highly expressed in AT vasculature.^[^ ^23^ ^]^ Furthermore, CD44 is expressed in both 3T3‐L1 and RAW264.7 cells.^[^ ^15^, ^24^ ^]^ Because P3 peptide binds specifically to PHB and HA binds specifically to CD44, we therefore investigated whether P3 peptide and HA could improve the uptake of our prepared nanosandwich in 3T3‐L1 and RAW264.7 cells. The optimal uptake time of P3‐HA/PM@BP was assessed by confocal laser scanning microscope (CLSM) and flow cytometry (FCM), which showed that its best cellular uptake time in both cells was 4 h (Figures S5 and S6, Supporting Information). In addition, PHB receptors in differentiated and undifferentiated 3T3‐L1 cells were immunolabeled with Cy3 after treatment with different NCs. As the CLSM images revealed, the red fluorescence of PHB was clearly observed in mature adipocytes only, indicating the expression of PHB on mature adipocyte cell membrane (**Figure** **3**a,c).^[^ ^23^ ^]^ We further observed a stronger green fluorescence of FITC‐labeled P3‐HA/PM@BP in differentiated 3T3‐L1 cells compared to that in undifferentiated cells, and the green fluorescence in P3‐HA/PM@BP group was stronger than that in HA/PM@BP group, indicating that the specific conjugation of P3 peptide to PHB receptor promoted the uptake of nanosandwich in adipocytes. Moreover, we also noticed a stronger green fluorescence in HA/PM@BP group compared to that in PM@BP or PolyMet groups in adipocytes and macrophages, suggesting that CD44 receptors facilitated the uptake of HA‐shelled NCs in both cells (Figure 3a,b,d,e).

Previous studies have demonstrated that HMW HA in free form or HA‐shelled NPs can poly‐cluster CD44, while low‐molecular‐weight (LMW) HA in free form disrupts this process.^[^ ^15^, ^25^ ^]^ Inspired by these findings, we then observed the changes of CD44 receptors on RAW264.7 and 3T3‐L1 cells after immune‐fluorescently staining with Cy3. As shown in Figure 3f, CD44 receptors were evenly distributed on the surface of both cells after PBS and LMW HA treatments. In contrast, after incubation with HA/PM@BP, some aggregated fluorescent dots appeared on one side of the cells, representing the formation of CD44 clusters, which was beneficial to inhibiting the downstream pathway of CD44 and contributed to the anti‐inflammation and lipid reduction effects. Meanwhile, P3‐HA/PM@BP could also cluster CD44 after the addition of rhMMP2, confirming that our prepared nanosandwich exposed HA and poly‐clustered CD44 receptors after breakage by enzyme.

### 2.4 Lipid Reduction Effect In Vitro

**Figure 4:**
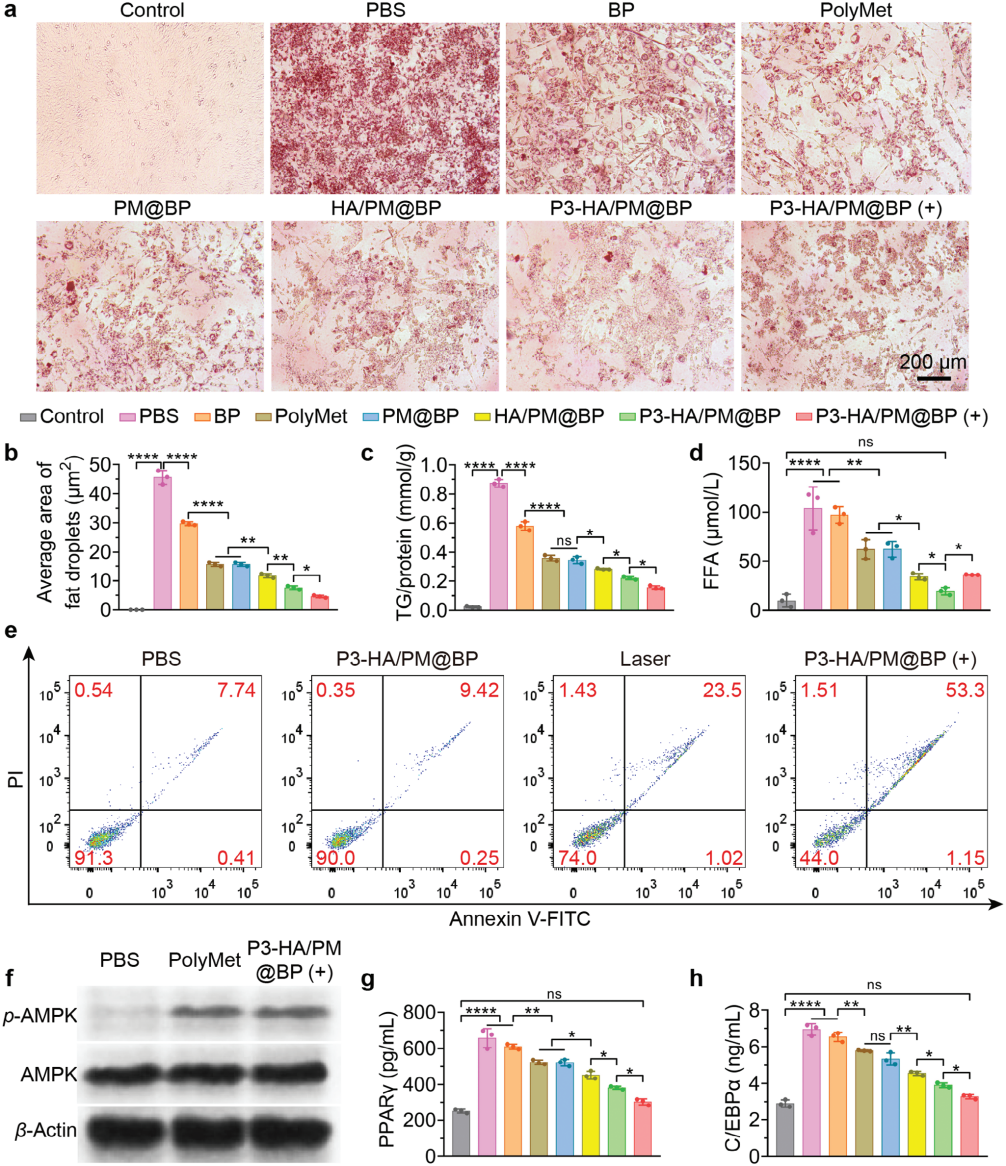
Lipid reduction effects of P3‐HA/PM@BP nanosandwich in vitro. a) Oil Red O staining images of 3T3‐L1 cells after different treatments. Scale bar = 200 µm. b) Average area of fat droplets in (a) quantified by Image J (*n* = 3). The levels of c) intracellular TG and d) released FFA were determined by colorimetric quantification kits (*n* = 3). e) 3T3‐L1 cells double‐stained with FITC‐Annexin V/PI after different treatments analyzed by FCM to distinguish apoptotic cells. f) Western blotting results of the protein expression of *p*‐AMPK in 3T3‐L1 cells after different treatments (*n* = 3). The quantified intracellular levels of g) PPARγ and h) C/EBPα using ELISA kits (*n* = 3). One‐way ANOVA followed by Tukey's multiple comparisons test was used for comparisons among multiple groups: ns indicated *p* > 0.05, ^*^ *p* < 0.05, ^**^ *p* < 0.01, ^****^ *p* < 0.0001.

The optimal concentration of P3‐HA/PM@BP to reduce lipid in 3T3‐L1 cells was determined by Oil Red O staining and MTT assay at a series of PolyMet concentrations, which showed that PolyMet could significantly decrease lipid droplets and had negligible cytotoxicity to 3T3‐L1 cells even at 0.02 mg mL^−1^ (Figure S7 and S8, Supporting Information). As illustrated by Oil Red O staining, the differentiated adipocytes in the PBS group had massive lipid droplets, which decreased significantly after PolyMet or PM@BP treatments along with reduced triglyceride (TG) and FFA (**Figure** **4**a–d; Figure S9, Supporting Information). As expected, BP also reduced the lipid contents in comparison to PBS treatment, which was presumably due to that the utilization of differentiation inducers was hindered by the absorption of BP to the cell surface. Besides, we found lower TG contents in the HA/PM@BP group than in the PM@BP group, demonstrating the lipid reduction effect of CD44 clustering by HA. Due to the P3 peptide‐mediated endocytosis, P3‐HA/PM@BP further reduced the lipid content in 3T3‐L1 cells, which was amplified after irradiation. Moreover, P3‐HA/PM@BP plus irradiation (P3‐HA/PM@BP (+)) resulted in the shrinkage of adipocytes and the virtual invisibility of intracellular lipid droplets, which was accompanied by an increased release amount of FFA. These results demonstrated that PTT can effectively induce lipolysis, which was consistent with the previous studies.^[^ ^3^, ^26^ ^]^ In addition, FCM analysis for apoptotic cell detection using Annexin V/PI double staining revealed that P3‐HA/PM@BP‐treated 3T3‐L1 cells showed 54.4% apoptotic cells after NIR laser irradiation, which was much higher than that without irradiation (Figure 4e). Taken together, adipocytes can be effectively damaged through PTT‐induced lipolysis and apoptosis.

Because MET can activate the AMPK signaling pathway, a key master regulator of cellular energy homeostasis and metabolism, to regulate lipid profile such as lipogenesis and FFA oxidation.^[^ ^27^ ^]^ We then investigated whether PolyMet, which contains a large number of biguanide groups, could also activate AMPK in 3T3‐L1 cells. The Western blotting results showed that a significant increase of the protein expression of phosphorylated AMPK (*p*‐AMPK) was identified in both PolyMet and P3‐HA/PM@BP (+) groups compared to the PBS group, confirming that PolyMet was also a regulator of AMPK signaling pathway and inherited the metabolic regulation from Met to modulate adipogenesis and lipogenesis (Figure 4f; Figure S10, Supporting Information). Furthermore, PPARγ and C/EBPα are key adipokines that induce adipocyte maturation and initiate adipogenesis and are the downstream pathways of AMPK.^[^ ^28^ ^]^ ELISA measurement showed that both the cellular contents of PPARγ and C/EBPα after different treatments decreased with a similar trend to TG, among which, P3‐HA/PM@BP (+) contributed to the most pronounced regulations on PPARγ and C/EBPα to restore their contents to normal levels (Figure 4g,h). These results indicated that P3‐HA/PM@BP nanosandwich plus laser treatment remarkably inhibited adipogenesis and lipogenesis in vitro due to the restoration of PPARγ and C/EBPα through AMPK.

### 2.5 Anti‐Inflammation Effect In Vitro

**Figure 5:**
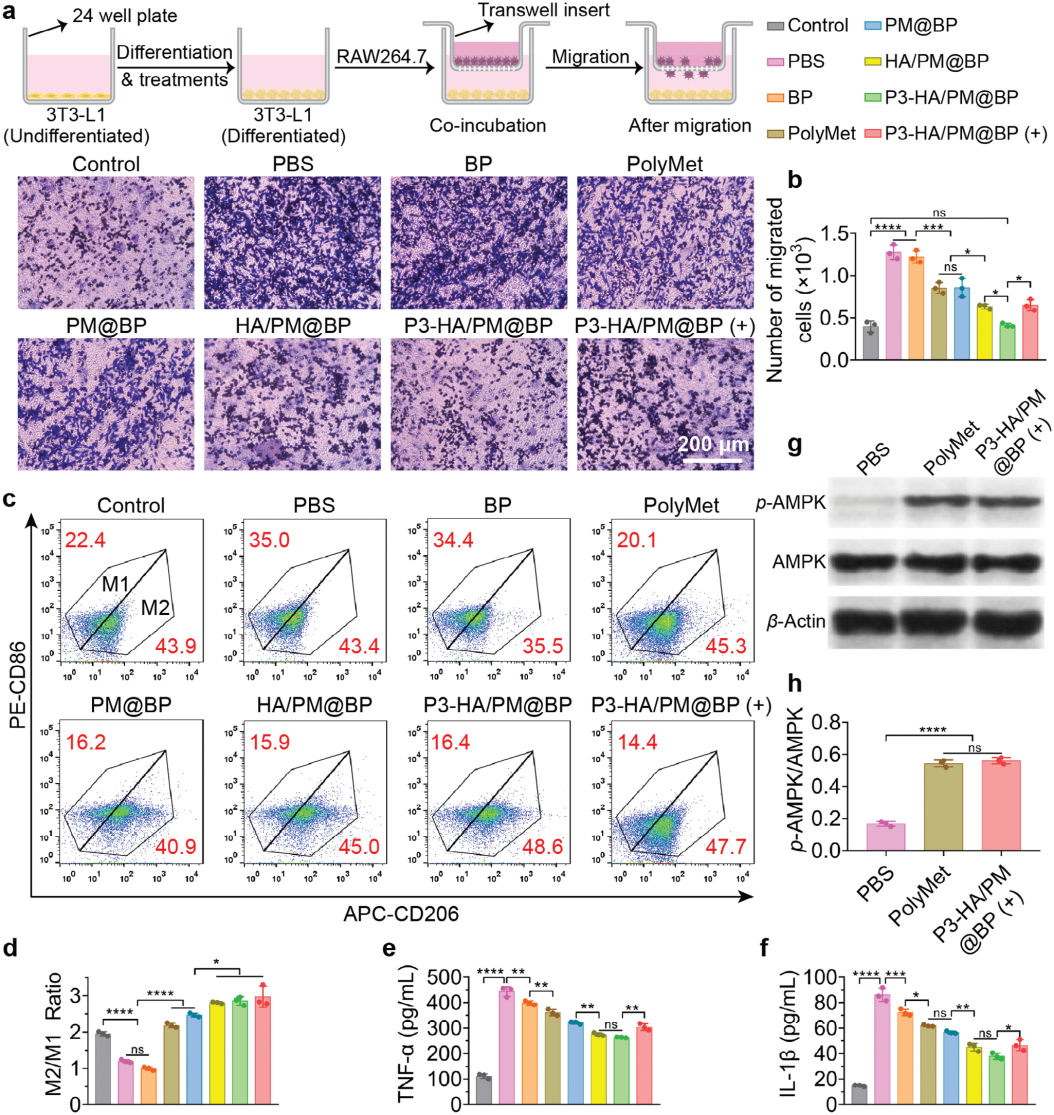
Anti‐inflammation effects of P3‐HA/PM@BP nanosandwich in vitro. a) Schematic demonstration of experiment procedure and results of macrophage migration assays. The migration of RAW264.7 cells was determined by a co‐cultured system of 3T3‐L1 and RAW264.7 cells in a transwell plate. b) Quantified number of migrated cells by Image J software (*n* = 3). c) Representative FCM plots and d) their quantified ratio of CD206^+^/CD86^−^ M2 to CD206^−^/CD86^+^ M1 cells after different treatments (*n* = 3). The levels of e) TNF‐α and f) IL‐1β in culture supernatant assessed by ELISA kits (*n* = 3). g) Western blotting results and h) the protein expression of *p*‐AMPK in RAW264.7 cells after different treatments (*n* = 3). One‐way ANOVA followed by Tukey's multiple comparisons test was used for comparisons among multiple groups: ns indicated *P* > 0.05, *P < 0.05, **P < 0.01, ***P < 0.001, ****P < 0.0001.

It has been shown that the enhanced MCP‐1 expression by adipocytes in obese AT could recruit large numbers of macrophages, which play key roles in triggering inflammatory response and exacerbating obesity.^[^ ^29^ ^]^ The influence of our prepared nanosandwich on macrophage chemotaxis toward hypertrophic adipocytes was investigated according to the transwell protocol indicated in **Figure** **5**a. RAW264.7 cells in the upper chamber of transwell plates were co‐incubated with differentiated 3T3‐L1 cells in the lower chamber and treated with different prepared formulations for 1 day. Then, the migrated RAW264.7 cells were visualized by staining with crystal violet and quantified accordingly, undifferentiated adipocytes were used as control (Figure 5b). It was obvious that macrophages tended to migrate to hypertrophic adipocytes, which increased by 2.22 times than that to immature adipocytes, while BP had little influence on macrophage migration. In addition, we observed that the mobility of RAW264.7 cells was reduced by 33.16% and 32.90% in the PolyMet and PM@BP groups respectively, which confirmed the anti‐migratory effect of PolyMet on macrophages. Moreover, HA/PM@BP and P3‐HA/PM@BP further inhibited their migration by 50.39% and 67.62%, respectively, which might be attributed to CD44 clustering by HA and enhanced P3‐mediated endocytosis. Unexpectedly, the number of migrated macrophages increased by 0.57 times after laser irradiation, which was not different from that in HA/PM@BP group. This phenomenon might be due to the fact that PTT first induced the lipolysis and apoptosis of 3T3‐L1 cells as shown in Figure 4d, e above, then, the intracellular nucleotides released by apoptotic cells and FFA generated from lipolysis served as potent inducers of macrophage migration.^[^ ^30^ ^]^ Macrophages in AT can be roughly categorized into two distinct subtypes, M1 and M2, in response to different microenvironmental factors. M1 macrophages express the surface marker CD86 and produce pro‐inflammatory cytokines such as TNF‐α, interleukin‐1beta (IL‐1β), and IL‐6. M2 macrophages express the surface marker CD206 and produce anti‐inflammatory cytokines such as IL‐10.^[^ ^31^ ^]^ To investigate the role of our designed nanosandwich on macrophage polarization, FCM was carried out to analyze the phenotypes of macrophages after different treatments. As illustrated in Figure 5c,d, PolyMet and PM@BP increased the proportion of CD206^+^CD86^−^ M2 macrophages and decreased the proportion of CD206^−^/CD86^+^ M1 macrophages, and consequently elevated the M2/M1 ratio significantly, which validated the repolarizing effect of PolyMet on macrophages.^[^ ^32^ ^]^ In addition, HA/PM@BP, P3‐HA/PM@BP, and P3‐HA/PM@BP (+) treatments further increased the M2/M1 ratios of RAW264.7 cells by 1.34, 1.38, and 1.48 times compared to PBS treatment, respectively, displaying effective pro‐M2 polarization effect. Pro‐inflammatory cytokines such as TNF‐α and IL‐1β overexpressed by macrophages in obese AT not only exacerbate obesity, but also trigger complications of atherosclerosis, nonalcoholic fatty liver disease (NAFLD), systemic insulin resistance, and even T2D.^[^ ^33^ ^]^ The concentrations of TNF‐α and IL‐1β in the supernatant of LPS‐polarized RAW264.7 cells after different treatments were determined (Figure 5e,f). As expected, the levels of these two cytokines were extremely low in the control group and increased significantly after LPS stimulation, while P3‐HA/PM@BP dramatically reduced the levels of TNF‐α by 40.52% and IL‐1β by 55.96%, which demonstrated the great anti‐inflammatory effects of our prepared nanosandwich in vitro. Unexpectedly, the contents of TNF‐α and IL‐1β increased by almost 0.16 and 0.23 times after laser irradiation, which might be attributed to photothermal‐induced RAW264.7 cell death, subsequently triggering an inflammatory signal for cytokine expression (Figure S11, Supporting Information).^[^ ^34^ ^]^

Since Met could relieve macrophage inflammatory responses by activating the AMPK signaling pathway, such as repolarizing M1 to M2, decreasing macrophage chemotaxis and inflammatory factors expression, we subsequently investigated whether the anti‐inflammatory efficacies exerted by PolyMet and P3‐HA/PM@BP (+) were also mediated by AMPK.^[^ ^35^ ^]^ As the result of Western blotting analysis implied, there were significantly higher protein expressions of *p*‐AMPK in PolyMet and P3‐HA/PM@BP (+) groups compared to the PBS group, confirming that PolyMet was engaged in the regulation of AMPK and contributed to inflammation alleviation (Figure 5g,h). In conclusion, we demonstrated the promising anti‐inflammatory effects of PolyMet and P3‐HA/PM@BP nanosandwich using macrophage as a reasonable model in vitro.

### 2.6 Anti‐Obesity Effect In Vivo

**Figure 6:**
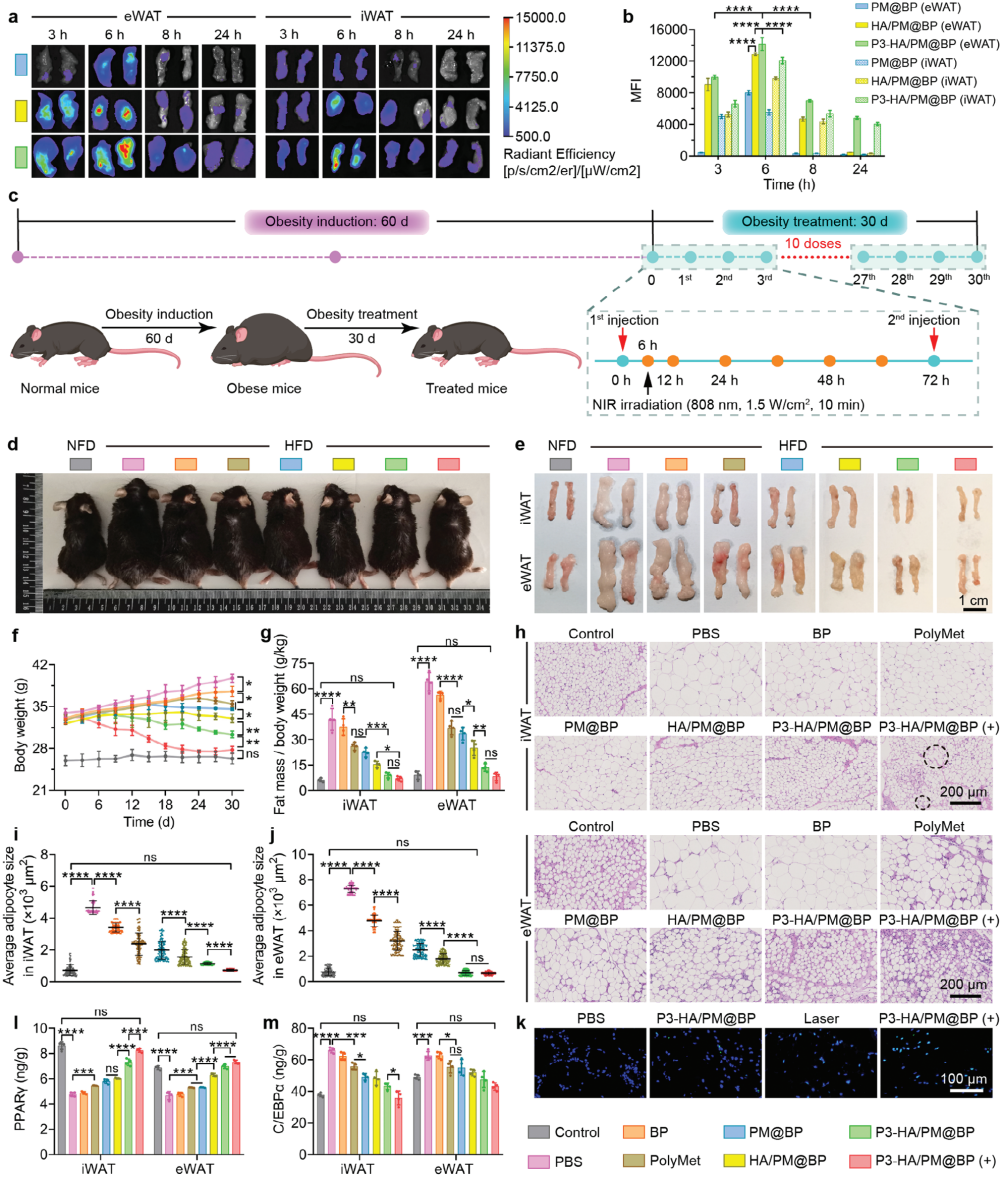
Anti‐obesity efficacies of P3‐HA/PM@BP nanosandwich in vivo. a) Representative fluorescence images and b) quantitative analysis of MFI of iWAT and eWAT at 3, 6, 8, and 24 h after injection of different NCs (*n* = 4). c) Timeline diagram of obesity model induction and treatment circle of anti‐obesity studies. Photographs of d) mice and e) excised iWAT and eWAT from different groups (Scale bar = 1 cm). f) Mice body weight and g) relative fat mass in different groups (*n* = 4). h) H&E‐stained images of iWAT and eWAT (Scale bar = 200 µm), where damaged adipocytes were surrounded by black dotted circles, and i,j) the corresponding adipocyte sizes evaluated by Image J. k) Apoptosis of adipocytes in iWAT indicated by TUNEL staining (green). Scale bar = 100 µm. The levels of l) PPARγ and m) C/EBPα in iWAT and eWAT were determined by ELISA kits (*n* = 4). One‐way ANOVA followed by Tukey's multiple comparisons test was used for comparisons among multiple groups: ns indicated *p* > 0.05, ^*^ *p* < 0.05, ^**^ *p* < 0.01, ^***^ *p* < 0.001, ^****^ *p* < 0.0001.

The anti‐obesity efficacy of P3‐HA/PM@BP nanosandwich in vivo was performed on diet‐induced obesity (DIO) mice fed with a high‐fat diet (HFD) containing 60% kcal from fat for 60 days to increase their body weight to ≈35 g. The successful establishment of the DIO model was also verified by the levels of body size, Lee's index, AT volume, adipocyte size, and the levels of PPARγ, C/EBPα, TNF‐α, and IL‐1β (Figure S12, Supporting Information). The targeting effect of nanosandwich to inguinal adipose tissue (iWAT) and epididymal adipose tissue (eWAT) was revealed by ex vivo images at specific time points after intravenous injection of Cy5‐labeled PM@BP, HA/PM@BP, and P3‐HA/PM@BP (**Figure** **6**a,b). The quantified MFI showed that a stronger fluorescence signal of Cy5 was observed in eWAT than iWAT at any time point for all three NCs, which may be related to the abundant blood vessel density in eWAT.^[^ ^36^ ^]^ Among the three NCs, P3‐HA/PM@BP exhibited the highest accumulation in both eWAT and iWAT owing to the dual‐targeting of P3 and HA, which facilitated the interaction between nanosandwich and AT, while the targeting effect of HA/PM@BP NCs was weaker because of the single‐targeting of HA. Notably, the accumulation of PM@BP in AT was the least because of its non‐specific distribution.

Furthermore, the power of the NIR laser was tested, which showed that 1.5 W cm^−2^ power was adequate to raise AT temperature to 46.7 ± 0.4°C, which was sufficient to induce apoptosis and lipolysis as well as prevent skin damage (Figure S13, Supporting Information).^[^ ^3b^ ^]^ The photothermal effect of P3‐HA/PM@BP nanosandwich after irradiation for 10 min was evidenced by the temperature rise from 33.6 ± 0.1 °C to 45.7 ± 0.4 °C (Figure S14, Supporting Information). Then, the in vivo anti‐obesity effects of P3‐HA/PM@BP in combination with PTT were performed in DIO mice according to the 30‐day dosing timeline (Figure 6c). Met and HA were reported to regulate obesity through inhibiting adipogenesis and lipogenesis, in consistency with those reported studies, we found notable improvements in mice body shape, AT volume, body weight, relative fat mass, and Lee's index in P3‐HA/PM@BP (+) group (Figure 6d–g; Figure S15a, Supporting Information). The stable food intake preliminarily confirmed the biocompatibility of nanosandwich, which further suggested that weight loss was attributed to the applied treatments rather than the reduction in energy intake (Figure S15b, Supporting Information). Subsequently, according to the results of H&E‐stained images, we noticed that adipocytes from normal mice were small, while adipocytes from obese mice were hypertrophic. In addition, we found more violet‐blue nuclear staining in eWAT than in iWAT, suggesting that visceral adipose tissue (VAT) is highly predictive of the morbidity of complications such as IR, T2D, and NAFLD due to the complex environment with more inflammatory and immune cells (Figure 6h).^[^ ^37^ ^]^ The average adipocyte sizes of iWAT or eWAT shrank to various degrees after different treatments, but P3‐HA/PM@BP (+) was the most effective, indicating its excellent lipid reduction effect (Figure 6i,j). Notably, the boundaries of adipocytes in iWAT were blurred or even disappeared after irradiation, which was displayed in black dotted circles, this phenomenon might be caused by the photothermal effect of BP. High‐temperature denatured cell membrane protein and disrupted the permeability of the membrane, leading to the influx of interstitial plasma and cell swelling. With the accumulation of heat, rupture of the cell membrane, and leakage of intracellular lipid droplets, the apoptosis of adipocytes finally happened.^[^ ^3b^ ^,26]^ TUNEL staining further verified these results (Figure 6k). Differently, eWAT did not show obvious apoptosis, which might be resulted from the penetration limitations of NIR (Figure S16, Supporting Information).^[^ ^38^ ^]^

PPARγ and C/EBPα are critical nuclear receptors in the pathogenesis of obesity, and PPARγ is inhibited and downregulated by increased TNF‐α under obese conditions.^[^ ^39^ ^]^ We evaluated the levels of PPARγ and C/EBPα in both iWAT and eWAT after different treatments (Figure 6l,m). PolyMet restored the contents of PPARγ and C/EBPα significantly, which may be attributed to the activation of AMPK. Further improved adipokine levels in the HA/PM@BP group suggested the adipogenesis inhibition effect of CD44 clustering by HA. P3‐HA/PM@BP regulated the adipokines contents most, especially after iWAT exposure to the laser, which illustrated the significance of dual‐targeting and PTT efficacy. In conclusion, P3‐HA/PM@BP (+) could inhibit adipogenesis and lipogenesis in vivo by restoring the levels of adipokines PPARγ and C/EBPα and inducing subcutaneous adipocyte apoptosis, ultimately reducing lipid contents in AT.

### 2.7 Anti‐Inflammation Effect and Blood Lipid Profiles In Vivo

**Figure 7:**
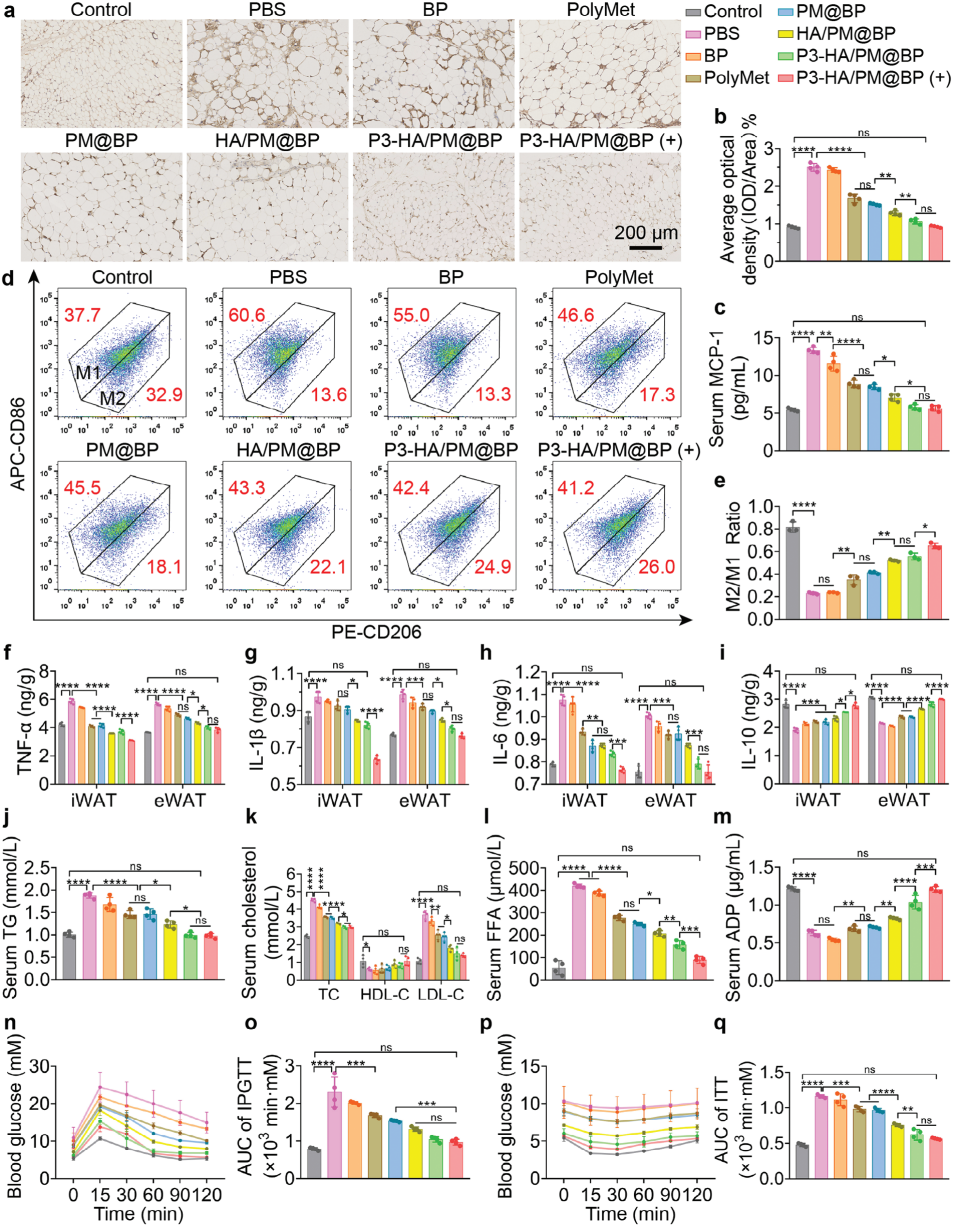
Anti‐inflammation effects and blood lipid profiles of P3‐HA/PM@BP nanosandwich in vivo. The effect of P3‐HA/PM@BP nanosandwich on macrophage chemotaxis determined by a) F4/80 immunostaining in eWAT dissected from all groups, and b) the quantified average optical density of F4/80‐positive cells (*n* = 4). Scale bar = 200 µm. The levels of c) MCP‐1 in serum were tested by ELISA (*n* = 4). d) Representative FCM plots and e) the quantified ratio of M2 macrophages to M1 macrophages after different treatments (*n* = 3). The levels of inflammatory factors including f) TNF‐α, g) IL‐1β, h) IL‐6, and i) IL‐10 in eWAT and iWAT determined by ELISA kits (*n* = 4). The serum lipid profiles of j) TG, k) cholesterol, l) FFA, and m) ADP (*n* = 4). n) The conducted intraperitoneal glucose tolerance test (IPGTT) and p) insulin tolerance test (ITT), and o,q) the calculated respective area under the curve (AUC) (*n* = 4). One‐way ANOVA followed by Tukey's multiple comparisons test was used for comparisons among multiple groups: ns indicated *p* > 0.05, ^*^ *p* < 0.05, ^**^ *p* < 0.01, ^***^ *p* < 0.001, ^****^ *p* < 0.0001.

It is found that the proportion of macrophages is positively correlated with adipocyte size and body weight, which is elevated in obese AT and involved in inflammation.^[^ ^40^ ^]^ We performed F4/80 staining to reveal macrophage chemotaxis to eWAT (**Figure** **7**a). The quantification of average optical density displayed that PolyMet reduced F4/80^+^ cells by 32.97%, indicating PolyMet was effective in reducing macrophage chemotaxis. Significantly, the numbers of macrophages after P3‐HA/PM@BP treatment with or without NIR irradiation were the least, demonstrating the effective dual‐targeting profile of P3 and HA in vivo (Figure 7b). A majority of macrophages in obese AT are differentiated from monocytes, which are recruited by MCP‐1.^[^ ^41^ ^]^ We found that there was a significantly decreased MCP‐1 level in eWAT and iWAT after treatments with NCs instead of free drugs, and there was a corresponding drop in MCP‐1 released into the bloodstream, which may contribute to the reduction of macrophage infiltration in AT (Figure 7c; Figure S17, Supporting Information). In addition, the FCM analysis of macrophages in eWAT revealed that P3‐HA/PM@BP (+) increased the proportion of F4/80^+^/CD86^−^/CD206^+^ M2 macrophages from 13.6% to 26.0%, and decreased the proportion of F4/80^+^/CD86^+^/CD206^−^ M1 macrophages from 60.6% to 41.2%, consequently increasing the ratio of M2/M1 by ≈2 times, which eventually contributed to inflammation relief (Figure 7d,e).

It has been revealed that obese AT contains higher levels of pro‐inflammatory cytokines, such as TNF‐α, IL‐1β, inducible nitric oxide synthase (iNOS), and IL‐6 compared to normal AT, which directly upregulate lipolysis and ectopic fat accumulation, and eventually lead to insulin resistance, diabetes or hypertriglyceridemia.^[^ ^9^, ^42^ ^]^ As shown in Figure 7f–i, the levels of pro‐inflammatory factors including TNF‐α, IL‐1β, and IL‐6 decreased, while the level of anti‐inflammatory factor IL‐10 increased after performed treatments, and P3‐HA/PM@BP nanosandwich exerted the optimal therapeutic outcomes in reshaping the cytokine levels in vivo. In addition, since IL‐6 is secreted by M1 and IL‐10 is released by M2, the increased IL‐6 and decreased IL‐10 levels also indicated the repolarization of macrophages from M1 to M2 in AT, which was consistent with the results of macrophage polarization assays in vivo. Therefore, the above results confirmed that PolyMet and P3‐HA/PM@BP nanosandwich could effectively alleviate inflammation in AT by reducing macrophage infiltration, repolarizing M1 to M2, increasing the secretion of anti‐inflammatory factors, and decreasing the expression of pro‐inflammatory factors.

The anti‐obesity effects were also examined by blood lipids. Among all the treatments, P3‐HA/PM@BP led to optimal improvements in the levels of TG, cholesterol, and FFA in serum (Figure 7j–l). P3‐HA/PM@BP (+) increased adiponectin (ADP) secretion from AT to serum, which may be facilitated by the increased PPARγ and favored systemic metabolism (Figure 7m; Figure S18, Supporting Information).^[^ ^43^ ^]^ Moreover, P3‐HA/PM@BP (+) treatment effectively lowered the fasting blood glucose level from 10.9 ± 1.4 mM to 5.7 ± 0.1 mM and facilitated blood glucose clearance and recovery after glucose or insulin injection (Figure 7n–q). The above results suggested that P3‐HA/PM@BP (+) could effectively alleviate obesity in a systemic way.

### 2.8 Transcriptome Analysis of eWAT After Treatment

**Figure 8:**
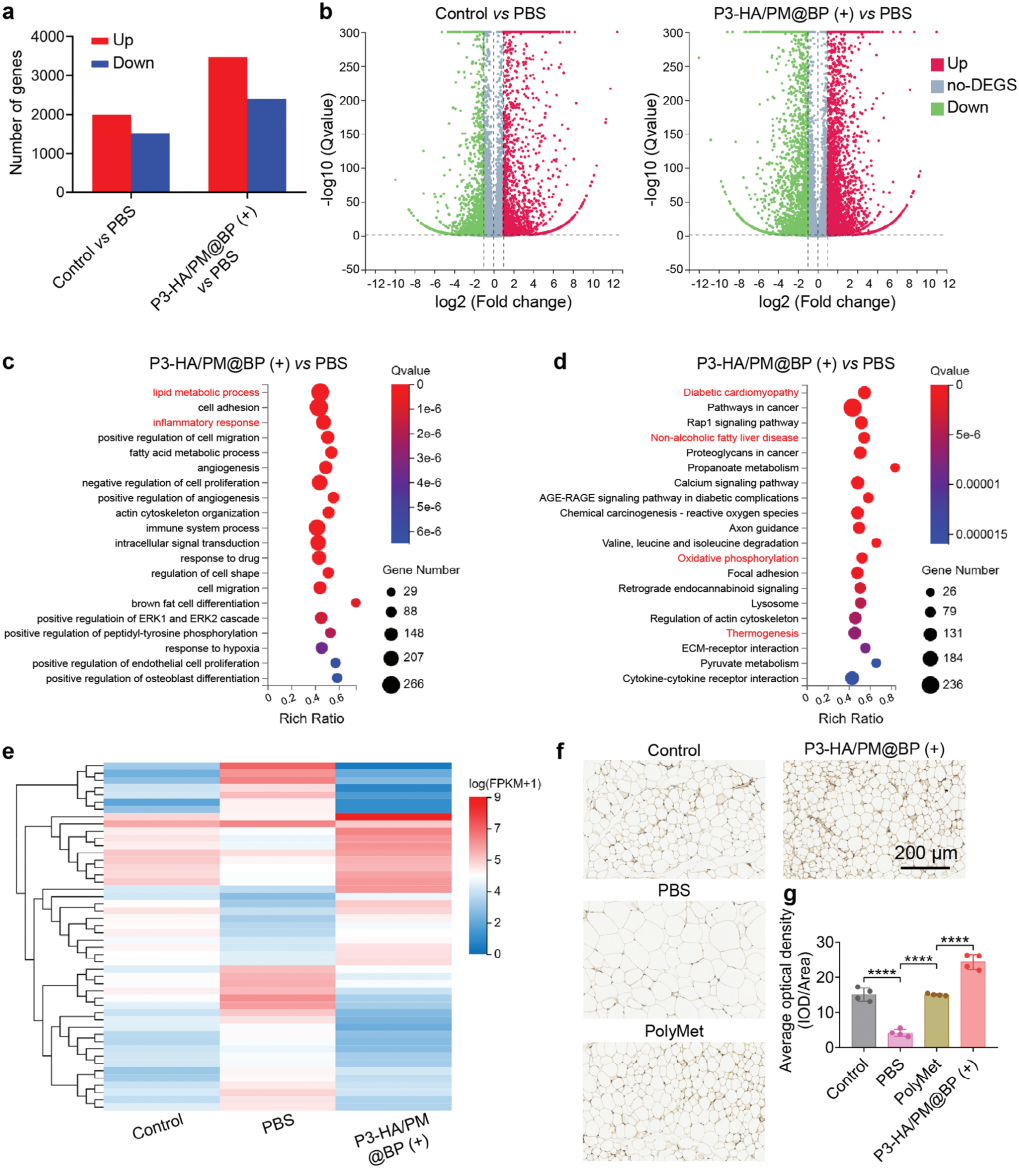
Transcriptome analysis of eWAT after P3‐HA/PM@BP nanosandwich treatment. The HFD‐fed obese mice received treatments of PBS, and P3‐HA/PM@BP (+) for 30 days, normal fat diet (NFD)‐fed mice were used as control. a) The number of DEGs in the (Control *vs* PBS) group and (P3‐HA/PM@BP (+) *vs* PBS) group. b) The volcano plots representing all DEGs in (Control *vs* PBS) group and (P3‐HA/PM@BP (+) *vs* PBS) group using red (up) and green (down) for each gene. c) GO biological processes enrichment analysis and d) KEGG signaling pathway analysis of all DEGs in the (P3‐HA/PM@BP (+) *vs* PBS) group. e) Hierarchical clustering of DEGs for comparisons among groups. The browning effect of PolyMet and P3‐HA/PM@BP (+) on eWAT characterized by f) anti‐UCP1 antibody staining, and g) the corresponding quantifications analyzed by Image J. One‐way ANOVA followed by Tukey's multiple comparisons test was used for comparisons among multiple groups: ^****^ *p* < 0.0001.

To further explore the therapeutic effects and mechanism of P3‐HA/PM@BP (+), we analyzed the transcriptomics of eWAT after different treatments. **Figure** **8**a showed a significant increase of 1995 genes and a significant decrease of 1517 genes in the PBS group compared to the control group, and a significant increase of 3479 genes and a significant decrease of 2401 genes in the PBS group compared to P3‐HA/PM@BP (+) group. The corresponding volcano plots of all differentially expressed genes (DEGs) are shown in Figure 8b. Based on the Gene Ontology (GO) and Kyoto Encyclopedia of Genes and Genomes (KEGG) signaling pathway enrichment analysis of all DEGs, the effects of P3‐HA/PM@BP (+) treatment on cell components were mainly concentrated in cytoplasm, mitochondrion, and cell surface (Figure S19a, Supporting Information). In addition, P3‐HA/PM@BP (+) was mainly associated with protein binding, actin binding, catalytic activity, and other molecular functions (Figure S19b, Supporting Information). Furthermore, P3‐HA/PM@BP (+) treatment participated in many biological processes, mainly including lipid metabolic process, cell adhesion, and inflammatory response, which confirmed the roles of our prepared nanosandwich in regulating lipid and inflammation (Figure 8c). Analysis of the KEGG signaling pathway showed that P3‐HA/PM@BP (+) was highly correlated with diabetic cardiomyopathy and NAFLD, whose morbidity and mortality developed in parallel with the increased incidence of obesity (Figure 8d).^[^ ^44^ ^]^ The clustering heat map showed that the gene expression levels of treated mice in the P3‐HA/PM@BP (+) group differed significantly from those of obese mice in the PBS group, whereas they were similar to those of normal mice in the control group, suggesting that P3‐HA/PM@BP plus laser irradiation could effectively restore gene expression to normal levels (Figure 8e). Additionally, DEGs were also significantly associated with browning‐related pathways, such as oxidative phosphorylation and thermogenesis shown in Figure 8d, thereafter, we performed uncoupling protein 1 (UCP1) staining to reveal brown adipose tissue (BAT) marker in eWAT (Figure 8f). After 30 days of P3‐HA/PM@BP (+) treatment, the level of UCP1 was 5.85 times higher than that in the PBS group, and PolyMet treatment was also effective in increasing the level of UCP1 by 2.61 times (Figure 8g). These results all demonstrated that PolyMet and P3‐HA/PM@BP (+) treatments were effective in promoting anti‐obesity, which was partly achieved through WAT browning.

### 2.9 Biosafety Assessments In Vivo

**Figure 9:**
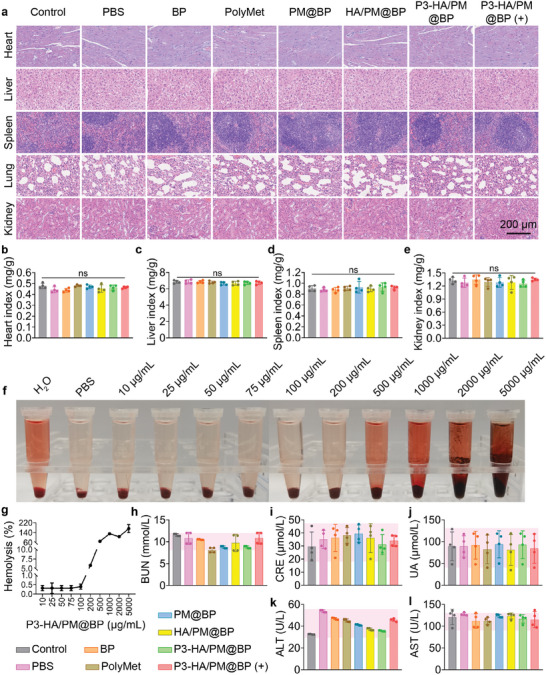
Safety assessments of P3‐HA/PM@BP nanosandwich in vivo. a) The H&E‐stained images of the heart, liver, spleen, lung, and kidney of C57BL/6J mice after different treatments. Scale bar = 200 µm. The indexes of b) heart, c) liver, d) spleen, and e) kidney (*n* = 4). f) Hemolysis assay of P3‐HA/PM@BP with different concentrations, and g) the calculated hemolysis ratio (*n* = 3). Blood biochemistry parameters of BUN h), CRE i), UA j), ALT k), and AST l) after different treatments (*n* = 4). One‐way ANOVA followed by Tukey's multiple comparisons test was used for comparisons among multiple groups: ns indicated *P* > 0.05.

The biocompatibility of P3‐HA/PM@BP nanosandwich was investigated by major organ histology and blood biochemistry of mice. After different treatments, there was no detectable necrosis, congestion, or hemorrhage in the heart, liver, spleen, lung, and kidney (**Figure** **9**a). In addition, the indexes of the heart, liver, spleen, and, kidney did not fluctuate significantly after different treatments, demonstrating that P3‐HA/PM@BP (+) would not cause obvious damage to major organs (Figure 9b–e). Moreover, incubation of P3‐HA/PM@BP up to 100 µg mL^−1^ with red blood cell suspensions did not show obvious hemolysis with a hemolysis ratio of less than 0.5%, which demonstrated the good hemocompatibility of P3‐HA/PM@BP nanosandwich during in vivo transportation (Figure 9f,g). Besides, all blood biochemical parameters of the kidney including blood urea nitrogen (BUN), creatinine (CRE), and uric acid (UA), and liver including alanine transaminase (ALT), and aspartate transaminase (AST) were within the normal range, which indicated that P3‐HA/PM@BP (+) caused negligible damage to kidney and liver (Figure 9h–l). Overall, all the investigations demonstrated that P3‐HA/PM@BP had desirable biocompatibility in vivo.

## 3 Conclusion

In conclusion, we developed a dual‐targeting, self‐assembled nanosandwich to achieve the goal of anti‐obesity treatment based on the strategy of “lipid reduction and anti‐inflammation”. P3‐HA/PM@BP nanosandwich could precisely target eWAT and iWAT due to the dual‐targeting of P3 and HA. Upon MMP2 enzymatic cleavage of the substrate linker in P3‐HA, the exposed HA poly‐clustered multiple CD44 on the surface of adipocytes and macrophages, eventually alleviating lipogenesis and inflammation. After cellular uptake, intracellular PolyMet activated the AMPK pathway, which subsequently reduced adipogenesis by regulating PPARγ and C/EBPα, and alleviated inflammation by inhibiting macrophage infiltration, repolarizing macrophage phenotype from M1 to M2, and decreasing the levels of pro‐inflammatory cytokines. Besides, BP exerted excellent photothermal effects after 808 nm laser irradiation, leading to the apoptosis of adipocytes and macrophages. Both in vivo and in vitro experiments demonstrated that there were significant reductions in adiposity and inflammation, and even AT browning was observed, all of which contributed to effective weight loss, improved insulin sensitivity and glucose tolerance without evident side effects after P3‐HA/PM@BP (+) treatment in DIO mice model. Collectively, this work proposes fresh insights in treating obesity based on the strategy of “lipid reduction and anti‐inflammation”, offering promising research value and clinical traceability.

## 4 Experimental Section

Materials, methods, preparation, and characterization of P3‐HA/PM@BP nanosandwich, in vitro and in vitro anti‐obesity studies are provided in Supporting Information.

### Animal Experiment

All animal experimental procedures were performed strictly following the National Institute of Health Guidelines for the Care and Use of Laboratory Animals and were approved by the Ethics Committee of China Pharmaceutical University (Ethics Code: 2021‐09‐014).

### Statistical Analysis

Statistical analysis was conducted using Prism 8.0 software (GraphPad Software, Inc.) and all data were expressed as mean ± SEM, indicated by error bars in all graphs. At least triplicate independent experiments were carried out unless otherwise noted. One‐way ANOVA followed by Tukey's multiple comparisons test was used for data analysis. *P* value < 0.05 was considered statistically significant (^*^ *p* < 0.05, ^**^ *p* < 0.01, ^***^ *p* < 0.001, ^****^ *p* < 0.001).

## Conflict of Interest

The authors declare no conflict of interest.

## Author Contributions

W.W., S.L., J.S., Q.X., and L.T. conceived and designed the experiments. Q.X., S.C., Y.M., C.W., and J.Y. performed the experiments. Q.X. and L.T. analyzed the data. Q.X. and L.T. wrote the manuscript. W.W., S.L., and J.S. edited the manuscript. W.W., S.L., J.S., Q.X., and L.T. involved in the discussion. W.W., S.L., and J.S. supervised the entire project. All authors discussed the results and commented on the manuscript.

## Supporting information



Supporting Information

## Data Availability

The data that support the findings of this study are available from the corresponding author upon reasonable request.
