# Supplementary material for: Two‐Pronged Attack: Dual Activation of Fat Reduction Using Near‐Infrared‐Responsive Nanosandwich for Targeted Anti‐Obesity Treatment
Source: Adv Sci (Weinh). 2024 Sep 26;11(43):2406985. doi: 10.1002/advs.202406985 (PMC11578330; doi:10.1002/advs.202406985)
Supplement: Supplementary file 1 — Supporting Information [file ADVS-11-2406985-s001.pdf]

ADVANCED SCIENCE
Open Access

Supporting Information

for *Adv. Sci.*, DOI 10.1002/advs.202406985

Two-Pronged Attack: Dual Activation of Fat Reduction Using Near-Infrared-Responsive Nanosandwich for Targeted Anti-Obesity Treatment

Qiaqia Xiao, Lu Tang, Siying Chen, Yijun Mei, Chuying Wang, Jing Yang, Jing Shang, Shengliang Li* and Wei Wang**

Supporting Information

Two-Pronged Attack: Dual Activation of Fat Reduction Using Near-Infrared-Responsive Nanosandwich for Targeted Anti-Obesity Treatment

Qiaqia Xiao[1,2], Lu Tang[1,2], Siying Chen[1,2], Yijun Mei[1,2], Chuying Wang[1,2], Jing Yang[1,2], Jing Shang[2,3], Shengliang Li[4*], Wei Wang[1,2*]*

[1] Q. Xiao, L. Tang, S. Chen, Y. Mei, C. Wang, J. Yang, W. Wang
State Key Laboratory of Natural Medicines, School of Pharmacy, China Pharmaceutical University, Nanjing 211198, P. R. China
E-mail: wangcpu209@cpu.edu.cn (W. Wang)

[2] Q. Xiao, L. Tang, S. Chen, Y. Mei, C. Wang, J. Yang, J. Shang, W. Wang
NMPA Key Laboratory for Research and Evaluation of Cosmetics, China Pharmaceutical University, Nanjing 211198, P. R. China
E-mail: shangjing21cn@cpu.edu.cn (J. Shang)

[3] J. Shang
School of Traditional Chinese Pharmacy, China Pharmaceutical University, Nanjing 211198, P.R. China

[4] S. Li
College of Pharmaceutical Sciences, Soochow University, Suzhou 215123, P. R. China
E-mail: lishengliang@suda.edu.cn (S. Li)

Experimental Section

Materials

Bulk black phosphorus (bulk BP) was purchased from Nanjing Xianfeng Nanomaterial Technology Co., Ltd. (Nanjing, CHN). Chitosan (CS), dicyandiamide, metformin (Met), potassium hexacyanoferrate (III), sodium nitroprusside, and glucose were purchased from Sinopharm Chemical Reagent Co., Ltd. (Shanghai, CHN). Hyaluronic acid (HA, molecular weight: 20 kDa) was supplied by Chengdu Ounreisi Chemical Reagent Co., Ltd. (Chengdu, CHN). P3-HA (HA-PLGLAG-CKGGRAKDC) was synthesized by CHNPeptides Co., Ltd. (Shanghai, CHN). 3-(4,5-dimethylthiazol-2-yl)-2,5-diphenyltetrazolium bromide (MTT) and fluorescein isothiocyanate (FITC) was purchased from Shanghai Aladdin Biochemical Technology Co., Ltd. (Shanghai, CHN). 4% paraformaldehyde, 4',6-diamidino-2-phenylindole (DAPI), crystal violet and lipopolysaccharide (LPS) were obtained from Beyotime Biotechnology Co., Ltd. (Shanghai, CHN). Sulfo-Cyanine3-NHS ester, and Sulfo-Cyanine5-NHS ester were purchased from Meilunbio Co., Ltd. (Dalian, CHN). Recombinant human matrix metalloproteinase 2 (rhMMP2) was purchased from Beijing Baiaolaibo Technology Co., Ltd. (Beijing, CHN). Immunoblotting antibodies: anti-phospho-AMPKα (Thr172) antibody, and anti-AMPKα1 antibody were purchased from Cell Signaling Technology Co., Ltd. (Boston, USA). Anti-β-actin antibody, Goat Anti-Rabbit IgG H&L (HRP), and Goat Anti-Mouse IgG H&L (HRP) were purchased from Abcam (Cambridge, UK). Immunohistochemical antibodies: anti-F4/80 biotin-conjugated primary antibody, anti-UCP1 biotin-conjugated primary antibody, and streptavidin-HRP secondary antibody were purchased from Servicebio Co., Ltd. (Wuhan, CHN). FCM antibodies: FITC-labeled anti-F4/80 antibody, APC-labeled anti-CD206 antibody, PE-labeled anti-CD86 antibody, PE-labeled anti-CD206 antibody, and APC-labeled anti-CD86 antibody were purchased from Elabscience Biotechnology Co., Ltd. (Wuhan, CHN). Immunofluorescent antibodies: Rabbit anti-PHB antibody was purchased from Proteintech (Wuhan, CHN), Rabbit anti-CD44 antibody and Cy3-labeled Goat anti-Rabbit secondary

antibody were obtained from Biocentury Co., Ltd. (Suzhou, CHN). Annexin V-FITC/PI cell apoptosis detection kit was obtained from Beyotime Biotechnology Co., Ltd. (Shanghai, CHN). Fetal bovine serum (FBS) was purchased from Thermo Fisher Scientific (Massachusetts, USA). Dulbecco's modified eagle medium (DMEM), 100 U/mL penicillin and 100 mg/mL streptomycin, trypsin-EDTA, and phosphate buffer solution (PBS, 0.1 M) were obtained from Nanjing SenBeijia Biological Technology Co., Ltd. (Nanjing, CHN). Dexamethasone (DEX), 3-isobutyl-1-methylxanthine (IBMX), insulin, and Oil Red O were purchased from Yuanye Biotechnology Co., Ltd. (Shanghai, CHN). Gelatin from cold water fish skin were purchased from Sigma-Aldrich (St. Louis, MO, USA). Mouse peroxisome proliferators-activated receptor gamma (PPARγ) and CAAT-enhancer-binding protein alpha (C/EBPα), adiponectin (ADP), monocyte chemotactic protein-1 (MCP-1), tumor necrosis factor alpha (TNF-α), interleukin-1beta (IL-1β), interleukin-6 (IL-6) and interleukin-10 (IL-10) enzyme linked immunosorbent assay (ELISA) kits were purchased from Biocloon Co., Ltd. (Nanjing, CHN). Triglyceride (TG), total cholesterol (TC), low-density lipoprotein cholesterol (LDL-C), low-density lipoprotein cholesterol (HDL-C), free lipid acid (FFA), aspartate transaminase (AST), alanine transaminase (ALT), blood urea nitrogen (BUN), creatinine (CRE), and uric acid (UA) assay kits were purchased from Nanjing Jiancheng Bioengineering Institute (Nanjing, CHN). High fat diet (HFD, 60% kcal from fat) was purchased from Xie Tong Pharmaceutical Bio-engineering Co. Ltd. (Nanjing, CHN). The other applied chemicals and reagents were commercially available in analytical level.

Preparation and characterization of PolyMet

PolyMet were synthesized by the addition reaction of hydrochloride chitosan (CS-HCl) and dicyandiamide as published before.[1] Briefly, CS-HCl was prepared by adding HCl to CS powder and then obtained after lyophilization. Subsequently, 1.5 g CS-HCl mixed with 0.1 M HCl solution was gradually heated to 100 °C, and 5.0 g dicyandiamide was added and reacted

for 3 h. After cooling the solution and separating the excess dicyandiamide, the supernatant was dialyzed (molecular weight cut off (MWCO) 3500 Da) in water. Finally, purified PolyMet was obtained after lyophilization.

To characterize the biguanide groups on PolyMet, FT-IR spectra was measured in the wave number range of 4000 ~ 400 cm^{-1} using potassium bromide pellet by infrared spectrometer (TENSOR 27, Bruker, GER), whereas free CS-HCl and Met were as control. UV-vis spectra and color test were applied to further characterize the biguanide groups. After dissolution in deuterium reagents (CF_3COOD : D_2O = 1:1 (v/v)), ^{13}C NMR spectra of PolyMet and CS-HCl were obtained by a NMR Spectrometer (AVANCE500, Bruker, 500 MHz).

Preparation and characterization of P3-HA/PM@BP nanosandwich

Firstly, BP was prepared by liquid phase exfoliation. Briefly, 15 mL acetone containing 15.0 mg bulk BP was ultrasonicated in a glass tube for 24 h using an ultrasonic probe (power, gradually increasing from 80 W to 200 W, on/off cycle, 2 s/2 s) (JY92-2D, Ningbo Scientz Biotechnology, CHN). The glass tube was maintained in an ice-water bath during ultrasonication. The mixture was then centrifuged (4000 rpm, 1 min, 25 °C, 5415R, Eppendorf, GER) to separate the unexfoliated bulk BP crystals, and the exfoliated BP was acquired after replacing the acetone solvent with pure water.

Subsequently, P3-HA/PM@BP nanosandwich was constructed with LbL electrostatic interaction. Briefly, 0.2 mg BP (equivalent to P content) was added dropwise into PolyMet solution (1 mL, 0.6 mg/mL) and kept stirring overnight at 25 °C. PM@BP nanocomplexes (NCs) were obtained after removing the excess PolyMet by ultrafiltration (MWCO 100 kDa, 3000 rpm, 30 min, 4 °C) and resuspension (power, 100 W, on/off cycle, 2 s/2 s, time, 10 min). Then, 0.2 mg PM@BP (equivalent to P content) was added dropwise to a mixture of HA (pH 8.0, 1 mL, 0.6 mg/mL) and P3-HA (50 μL, 1.2 mg/mL) and maintained stirring for 4 h at 25 °C. The mixture was then purified by high-speed centrifugation (13200 rpm, 20 min, 4 °C) to remove

the free HA and P3-HA, and redispersed in 1 mL pure water, followed by sonication (power, 80 W, on/off cycle, 2 s/2 s, time, 8 min) to obtain the final P3-HA/PM@BP nanosandwich. HA/PM@BP NCs were fabricated using the same method in the absence of P3-HA.

The size and zeta potentials of BP, PM@BP, HA/PM@BP and P3-HA/PM@BP were measured by dynamic scattering laser particle size (DLS) analyzer (NanoBrook Omin, Brookhaven, USA, 25 °C with 90 ° scattering angle). The chemical composition of the samples was confirmed by Raman spectra (DXR, Thermofisher, US). TEM (H-7650, Hitachi, JPN, 80 kV) and SEM (Sigma 300, Zeiss, GER) were used to observe the morphologies of pristine BP, PM@BP, and P3-HA/PM@BP nanosandwich. The formation of P3-HA/PM@BP was further characterized by fluorescent co-localization experiments. P3-HA/PM@BP nanosandwich was constructed with Cy3 labeled P3 peptide (Cy3-P3) and FITC labeled PolyMet (FITC-PM), and the fluorescence overlap were observed by laser confocal microscopy (LSM 700, Carl Zeiss, GER).

The drug loading efficiency (DLE) of PolyMet was determined by the content of free PolyMet in supernatant using UV-vis spectrum (UV756CRT, YOKE INSTRUMENT, CHN), and calculated by the below equation:

$$DLE = \frac{C-B}{A} \times 100\% \qquad \text{Equation (1)}$$

where A represented the mass of lyophilized P3-HA/PM@BP nanosandwich, B represented the mass of free PolyMet in supernatant, and C represented the mass of PolyMet added during preparation.

The stability and drug release profiles of P3-HA/PM@BP nanosandwich *in vitro*

The dispersion stability of P3-HA/PM@BP nanosandwich was evaluated by the particle size of prepared nanosandwich after incubation in pure water, PBS (pH 7.4), and DMEM containing 10% FBS at 4 °C for 1 week, respectively. The photothermal stability of P3-HA/PM@BP nanosandwich was evaluated by temperature change and NIR thermographs of BP (100 μg/mL)

and P3-HA/PM@BP in DMEM containing 10% FBS upon NIR laser irradiation (808 nm, 1.5 W/cm^2, 10 min) within one week, and temperature profiles upon 5 repeated on/off laser irradiation cycles on the 7th day.

The release profiles of PolyMet from P3-HA/PM@BP nanosandwich were evaluated by dialysis method. 2 mL P3-HA/PM@BP were dispersed in dialysis sacs (MWCO 1000 kDa) either in the presence or absence of 10 nM rhMMP2 at 37 °C. Then, dialysis sacs were soaked into 50 mL of PBS (pH 7.4) and placed on a shaker incubator (100 rpm, 37 °C). 2 mL samples were collected at specified time (0, 1, 2, 3, 4, 6, 8, 12, 18, and 24 h), followed by supplementing equal volume of PBS (pH 7.4). The release of PolyMet was measured by the absorption at 233 nm using UV-vis spectrum (UV756CRT, YOKE INSTRUMENT, CHN).

Cell culture

Murine fibroblast cell line 3T3-L1 and murine macrophage line RAW264.7 was obtained from Institute of Biochemistry and Cell Biology, Chinese Academy of Sciences (Shanghai, China) and were maintained in basic medium (DMEM with 10% FBS (v/v), 100 U/mL penicillin, and 100 μg/mL streptomycin) at 37 °C under humidified atmosphere containing 5 % CO_2.

To differentiate into mature adipocytes, 3T3-L1 cells were incubated in 6-well plates precoated with 0.1% gelatin (w/v) and cultured in basic medium to confluency for at least 2 days. Then, cells were cultured in differentiation medium (DMEM supplemented with 10% FBS (v/v), 100 U/mL penicillin, 100 μg/mL streptomycin, 10 μg/mL insulin, 1 μg/mL dexamethasone (DEX), and 5 mM isobutyl methylxanthine (IBMX)) for 4 days. After that, cells were further incubated with maintenance medium (DMEM supplemented with 10% FBS (v/v), 100 U/mL penicillin, 100 μg/mL streptomycin, and 10 μg/mL ISN) for 4 days, followed by incubation for another 2 days in basic medium to complete the differentiation.

Determination of the treatment concentration of PolyMet on 3T3-L1 cells

3T3-L1 preadipocytes were incubated in 6-well plates precoated with 0.1% gelatin and cultured in basic medium to confluency for at least 2 days. Then, cells were treated with a series of concentrations of PolyMet (0.00, 0.01, 0.02, 0.05, 0.10, and 0.20 mg/mL) in differentiation medium for 4 days. After that, cells were further treated with maintenance medium for 4 days, followed by incubation for another 2 days in basic medium. The intracellular lipid droplets were stained with Oil Red O (Shanghai Yuanye, CHN) and imaged using an inverted microscope (XD202, Jiangnan novel optics, CHN). The average area of fat drops dyed with Oil Red O were counted using Image J (NIH) software.

MTT assay

The MTT assay was used to assess the cell viability of 3T3-L1 cells exposed to PolyMet. 3T3-L1 cells were differentiated in 96-well plate and treated with a series of concentrations of PolyMet (0.00, 0.01, 0.02, 0.05, 0.10, and 0.20 mg/mL) as mentioned above. 3T3-L1 cells without treatment were used as control group, medium without cells was used as blank group. Then, 0.5% MTT were added to each well and cells were incubated for another 4 h at 37 °C. After that, 100 μL of DMSO were added and the cell viability was determined by measuring the optical density (OD) at 490 nm using a microplate reader (ELX800, Zeiss, GER). The viabilities of 3T3-L1 cells were calculated according to this equation:

$$\text{Cell viability (\%)} = \frac{OD_{Sample} - OD_{Blank}}{OD_{Control} - OD_{Blank}} \times 100\% \qquad \text{Equation (2)}$$

where OD_{Sample}, OD_{Blank}, and $OD_{Control}$ were the absorbance of sample, blank group, and control group, respectively.

Cellular uptake of 3T3-L1 and RAW264.7 cells

To investigate how treatment time influenced on the cellular uptake of P3-HA/PM@BP nanosandwich, 3T3-L1 cells and RAW264.7 cells were exposed to 30 μg/mL (equivalent to P

content) P3-HA/PM@BP prepared by FITC-PolyMet (FITC-nanosandwich) for different time period (1, 2, 4, and 6 h), followed by FCM analysis (Celesta, BD, USA). Cells were also stained with DAPI for CLSM analysis (LSM700, Carl Zeiss, GER).

To investigate the cellular uptake of different NCs, undifferentiated or differentiated 3T3-L1 cells and RAW264.7 cells were exposed to 30 μg/mL (equivalent to P content) FITC labeled PM@BP, HA/PM@BP, and P3-HA/PM@BP for 4 h, and FITC-PolyMet was used as control. Then, 3T3-L1 cells were incubated with Rabbit anti-PHB primary antibody (1:200, Proteintech, CHN) and Cy3-labeled Goat Anti-Rabbit secondary antibody (1:100, Biocentury, CHN), followed by FCM analysis (Celesta, BD, USA). Cells were also stained with DAPI for CLSM analysis (LSM700, Carl Zeiss, GER).

Poly-clustering CD44 receptor on 3T3-L1 and RAW264.7 cells

3T3-L1 preadipocytes were incubated in confocal dish precoated with 0.1% gelatin and differentiated into mature adipocytes as described above. RAW264.7 cells were incubated in confocal dish and treated with LPS (1 μg/mL) for 24 h. After that, both cells were exposed to 1 mL of DMEM containing PBS, free HA (Mw = 20 kDa, 125 μg/mL), HA/PM@BP (30 μg/mL, equivalent to P content) or P3-HA/PM@BP (30 μg/mL, equivalent to P content) plus rhMMP2 (10 nM) for another 2 h, respectively. After exposure, cells were fixed with 4% paraformaldehyde and incubated with Rabbit anti-CD44 primary antibody (1:50, Biocentury, CHN) and Cy3-labeled Goat Anti-Rabbit secondary antibody (1:100, Biocentury, CHN), then, cells were stained with DAPI for CLSM analysis (LSM700, Carl Zeiss, GER).

Lipid reduction effect of P3-HA/PM@BP (+) on 3T3-L1 cell

3T3-L1 cells were differentiated and treated with 30 μg/mL (equivalent to P content) BP, PM@BP, HA/PM@BP, and P3-HA/PM@BP, 0.02 mg/mL PolyMet, and PBS at the time of induction with differentiation medium. At the last day of differentiation, half of the cells treated with P3-HA/PM@BP were exposed to irradiation (808 nm, 1.5 W/cm^2) for 10 min. After 12 h,

apoptotic cells were stained with Annexin V-FITC/PI and identified by FCM (Celesta, BD, USA). Released FFA and intracellular TG was analyzed using colorimetric quantification kits (Nanjing Jiancheng, CHN). The intracellular protein levels of PPARγ and C/EBPα were quantified using ELISA kits (Biocloon, CHN). The intracellular lipid droplets were stained with Oil Red O (Shanghai Yuanye, CHN) and imaged using an inverted microscope (XD202, Jiangnan novel optics, CHN). The average area of fat drops stained with Oil Red O was counted using Image J (NIH) software. Finally, Oil Red O in each well was extracted by isopropanol and the absorbance at 510 nm was determined by microplate reader (ELX800, Biotek, USA).

Anti-inflammation effect of P3-HA/PM@BP (+) on RAW264.7 cell

RAW264.7 cells were seeded in 6-well plates and incubated with DMEM supplemented with 30 μg/mL (equivalent to P content) of BP, PM@BP, HA/PM@BP, and P3-HA/PM@BP, 0.02 mg/mL of PolyMet, and PBS for 24 h. Thereafter, half of the cells treated with P3-HA/PM@BP were exposed to irradiation (808 nm, 1.5 W/cm^2, 10 min), followed by being cultured with 1 μg/mL LPS for another 24 h except for the control group. Then, cells were harvested and stained with APC-labeled anti-CD206 antibodies (1:20 dilution, Elabscience, CHN) and PE-labeled anti-CD86 antibodies (1:20 dilution, Elabscience, CHN) for polarization analysis or stained with Annexin V-FITC/PI for apoptosis analysis. The extracellular levels of TNF-α and IL-1β were analyzed using ELISA kits (Biocloon, CHN). A co-culture transwell system of RAW264.7 and 3T3-L1 cells was carried out for macrophage chemotaxis assay. 3T3-L1 cells were differentiated and treated differently in 24-well plate and undifferentiated cells were used as control. Then, RAW264.7 cells suspended in serum-free DMEM were placed in the upper chamber with a pore size of 8 μm (NEST, CHN) and co-cultured for 24 h, after which, RAW264.7 cells in the chamber were fixed and stained with 0.2% crystal violet. After scraping off cells remained in the upper chamber, macrophages appeared on the bottom of the filter were observed and counted as chemotactic cells by image J software.

Western blotting analysis

After treatments with PBS, PolyMet and P3-HA/PM@BP (+), 3T3-L1 cells and RAW264.7 cells in 6-well plates were collected and resuspended in RIPA lysis buffer on ice for 20 min. Vortex the suspension for 2 min followed by centrifugation (14000 rpm, 20 minutes) to collect proteins free of lipid and cellular debris.

After gel electrophoresis and membrane transfer, the membrane was then blocked for 2 h at room temperature, and incubated with anti-phospho-AMPKα (Thr172) antibody (*p*-AMPK, 1:1000 dilution, Cell Signaling, USA), anti-AMPKα1 antibody (AMPK1α, 1:1000 dilution, Cell Signaling, USA), and anti-β-actin antibody (1 μg/ml, Abcam, USA) at 4 °C overnight. Then, the membrane was incubated with Goat Anti-Rabbit IgG H&L (HRP) (1:2000 dilution, Abcam, USA), and Goat Anti-Mouse IgG H&L (HRP) (1:2000, Abcam, USA) for 1 h at room temperature. Protein bands were imaged and the gray values of protein bands were analyzed using Image Pro Plus 6.0 software.

Establishment of DIO mice model

Male C57BL/6J mice (4 weeks, 12~14 g) were purchased from Animal Center of Yangzhou University (Yangzhou, CHN) and housed at (22 ± 1) °C with 12 h light/dark cycles. Mice were with free access to water and diet.

To establish DIO mice models, mice were fed with HFD (XTHF 60, 60% kcal from fat; Xie Tong, CHN) *ad libitum* for 60 days. Mice fed with NFD (Qing Long Shan, CHN) were used as control. To evaluate the degree of obesity, body shape, body weight, Lee' index (Lee' index = (body weight (g) × 1000)$^{1/3}$/ body length (cm)), AT volume, adipocyte size, and the expression levels of PPARγ, C/EBPα, TNF-α, and IL-1β in iWAT and eWAT were analyzed.

Targeting effect of P3-HA/PM@BP nanosandwich to WAT

C57BL/6J mice were intravenously injected with 5 mg/kg (equivalent to P content) PM@BP, HA/PM@BP, and P3-HA/PM@BP prepared by Cy5-layeled PolyMet (Cy5-PM), and the

iWAT and eWAT were harvested at 3, 6, 8, and 24 h after administration. The accumulation of NCs in iWAT and eWAT were visualized by an imaging system (IVIS Spectrum, PerkinElmer, USA) and the mean fluorescence intensity (MFI) was quantitatively analyzed by Living Image® software.

Photothermal effect of P3-HA/PM@BP nanosandwich *in vivo*

Obese mice were intravenously injected with 5 mg/mL P3-HA/PM@BP nanosandwich, followed by 808 nm laser irradiation at the inguinal region with different power (0.5, 1.0, 1.5, and 2.0 W/cm^2) for 10 min at 6 h post-injection. The temperature profile during irradiation was recorded by NIR thermograms (HIKVISION, CHN).

Obese mice were intravenously injected with PBS, and 5 mg/mL P3-HA/PM@BP nanosandwich. After 6 h post-injection, half of the mice in each group were irradiated (808 nm, 1.5 W/cm^2, 10 min) at the inguinal region, and the temperature profiles during irradiation were recorded by NIR thermographic camera (HIKVISION, CHN).

Anti-obesity effect of P3-HA/PM@BP (+) *in vivo*

The DIO mice were randomly divided into different treatment groups: intravenous injection with PBS as control, BP, PM@BP, HA/PM@BP, and P3-HA/PM@BP. The dosage of BP in each NCs was 5 mg/kg (equivalent to P content), the dosage of PolyMet in each NCs was 2.7 mg/kg of PolyMet. Each mouse was administered every 3 days for consecutive 30 days (10 doses). Mice in NIR laser group were irradiated (808 nm, 1.5 W/cm^2, 10 min) at the inguinal region 6 h post-injection. During treatments, body weight and food intake were monitored every 3 days. At the end of treatments, insulin sensitivity and glucose tolerance were analyzed through IPGTT and ITT. Mice were anaesthetized to measure the body length and take pictures of the body shapes. Then, mice were euthanized, blood samples, and adipose tissues (iWAT, eWAT) were collected for analyses.

The serum levels of TG, TC, LDL-C, HDL-C and FFA were determined using colorimetric quantification kits (Nanjing Jiancheng, CHN). The contents of PPARγ, C/EBPα, ADP, MCP-1, TNF-α, IL-1β, IL-6 and IL-10 in WATs, and the levels of ADP and MCP-1 in serum were measured by ELISA assay kits (Biocloon, CHN). iWAT and eWAT were sectioned for H&E staining. Moreover, eWAT was labeled with anti-F4/80 antibody (1:500-1000, Servicebio, CHN) for macrophage chemotaxis analysis, and immune-stained with anti-UCP1 antibody (1:500-1000, Servicebio, CHN) for browning evaluation. Apoptotic cells in iWAT and eWAT were evaluated by TUNEL.

Polarization assay of macrophages *in vivo*

FCM was performed to confirm the polarization of macrophages in eWAT. Briefly, eWAT isolated from mice were rinsed in PBS, cut into small pieces of 3-5 mm, and digested for 1 h in Hank's buffer containing type 1 collagenase (1 mg/mL, Sigma-Aldrich, USA). Then, EDTA was added into eWAT to allow incubation for another 10 min. Afterwards, the eWAT were passed through a mesh (100 μm) and fractionated by centrifugation (500 g, 10 min, 4 °C). Afterwards, 0.5 mL of erythrocyte lysate were added to eliminate red blood cells. Isolated adipocytes were centrifuged (500 g, 10 min, 4 °C) and resuspended in FCM buffer with concentration of 2×10^6 cell/mL after counting. Cells were incubated in dark with FITC-labeled anti-F4/80 antibody (1:20 dilution, Elabscience, CHN), APC-labeled anti-CD86 antibody (1:20 dilution, Elabscience, CHN) and PE-labeled anti-CD206 antibody (1:20 dilution, Elabscience, CHN), followed by FCM analysis.

Transcriptome analysis

Total RNAs were exacted from eWAT that were derived from three independent mice: normal mice fed with NFD and treated with PBS (Control), obese mice fed with HFD and treated with PBS (PBS) or P3-HA/PM@BP (+). |log2 fold change| > 1 and Q values < 0.05 were set as the cutoff criteria, and DEGs between PBS and P3-HA/PM@BP (+) treated mice were quickly

analyzed using the Dr. Tom, an online software developed by Beijing Genomic Institute (BGI) (https://biosys.bgi.com/), with the GO and KEGG databases.

Biocompatibility and safety evaluation *in vivo*

After different treatments, mice were euthanized, major organs (heart, liver, spleen, lung and kidney) and blood samples were collected. Organs were weighed, and sectioned for H&E staining and observed under Slice Scanner (NanoZoomer 2.0 RS, Hamamatsu, JPN). The serum levels of BUN, UA, CRE, ALT, and AST were measured by colorimetric quantification kits (Sigma-Aldrich, USA).

Hemolysis assay

2 mL of fresh mouse blood were collected into an anticoagulant tube followed by centrifugation at 2000 rpm for 10 min to obtain the erythrocyte precipitate. Erythrocytes were then washed with PBS (pH 7.4) several times until the supernatant was clear and transparent. Subsequently, 2% erythrocyte suspension was prepared by gently dispersing the erythrocytes in PBS. Separately, 0.5 mL of P3-HA/PM@BP dispersed in PBS with different concentrations (10, 25, 50, 75, 100, 200, 500, 1000, 2000, and 5000 μg/mL) was added to 1.5 mL centrifuge tubes, and then 1 mL of erythrocyte suspension was added and co-cultured at 37 °C for 1 h. Erythrocyte suspension were co-cultured with PBS as a negative control group, and co-cultured with pure water as a positive control group. After that, the samples were then centrifuged at 3500 rpm for 10 min and the OD value of the supernatant was measured at 545 nm, the hemolysis ratio was calculated as follows:

$$\text{Hemolysis (\%)} = \frac{OD_{sample} - OD_{negative}}{OD_{positive} - OD_{negative}} \times 100 \qquad \text{Equation (3)}$$

where OD_{sample}, $OD_{negative}$ and $OD_{positive}$ were the absorbance of the sample, negative control, and positive control, respectively.

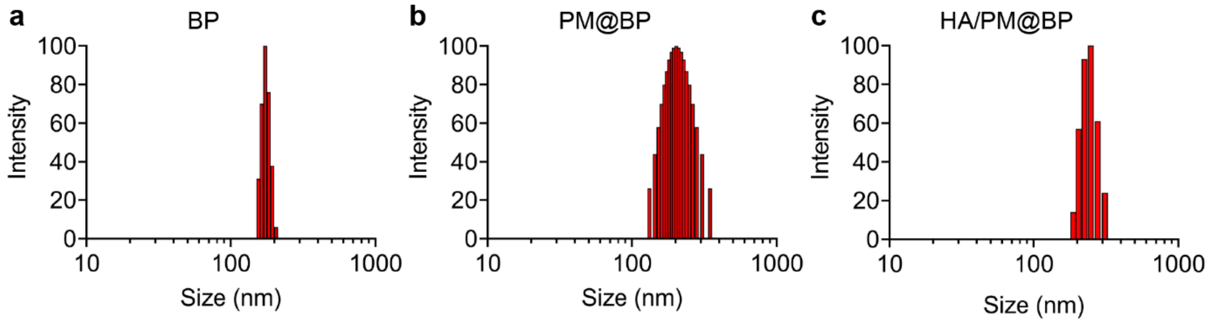

Figure S1. Hydrodynamic diameters of (**a**) BP, (**b**) PM@BP and (**c**) HA/PM@BP NCs.

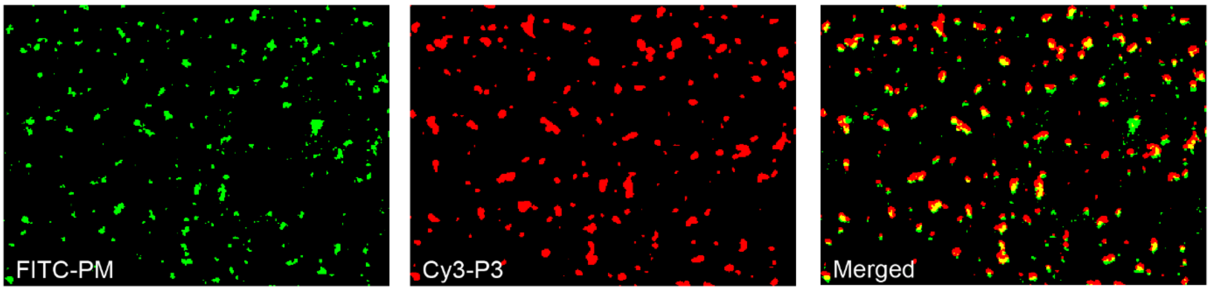

Figure S2. Fluorescence co-localization of P3-HA/PM@BP nanosandwich. FITC-PM and Cy3-P3 were used to replace PolyMet and P3 peptide to construct P3-HA/PM@BP nanosandwich, respectively.

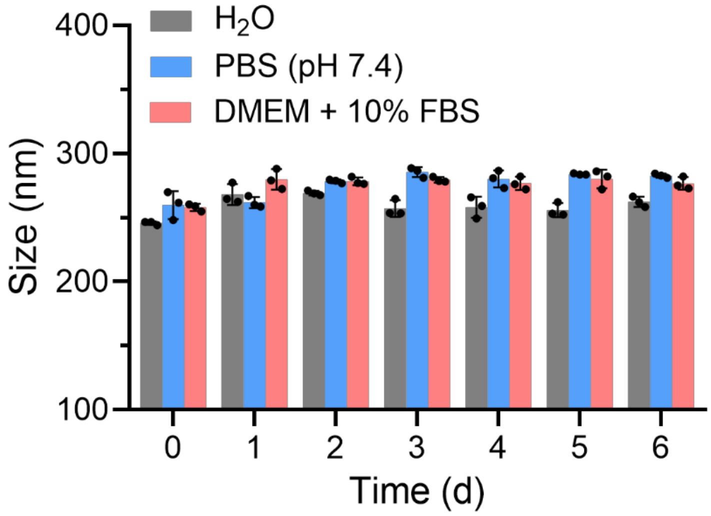

Figure S3. Stability of P3-HA/PM@BP nanosandwich in various media. The hydrodynamic diameter of P3-HA/PM@BP dispersed in pure water, PBS (pH = 7.4), and DMEM containing 10% FBS within one week (n = 3).

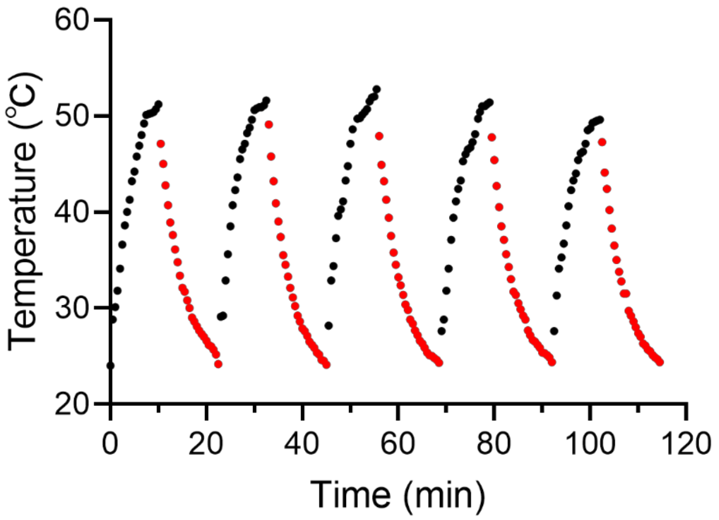

Figure S4. Photothermal stability of P3-HA/PM@BP nanosandwich. Temperature profiles of P3-HA/PM@BP (100 μg/mL) in DMEM containing 10% FBS upon 5 repeated on/off NIR laser irradiation cycles (808 nm, 1.5 W/cm^2, 10 min irradiation in each cycle), where black meant laser on, and red meant laser off.

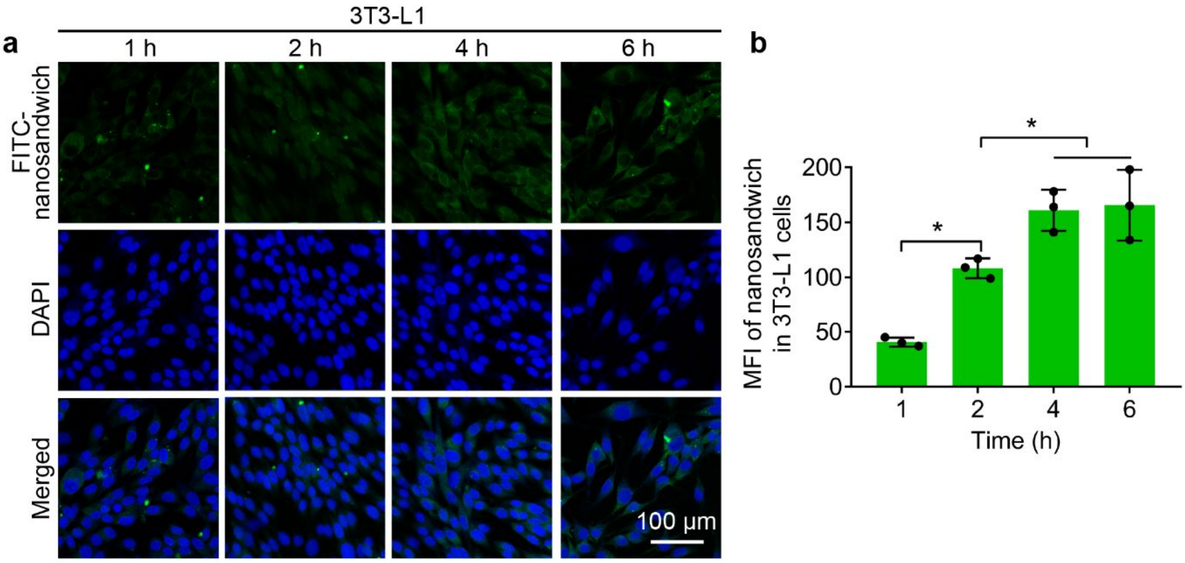

Figure S5. Cellular uptake of P3-HA/PM@BP nanosandwich by 3T3-L1 cells after different treatment time. (**a**) CLSM images of 3T3-L1 cells treated with 30 μg/mL of FITC-labeled P3-HA/PM@BP nanosandwich (green) for different time, the nuclei were stained with DAPI (blue). Scale bar = 100 μm. (**b**) Mean fluorescence intensity (MFI) of internalized nanosandwich in 3T3-L1 cells quantified by flow cytometry (FCM) (n = 3). One-way ANOVA followed by Tukey's multiple comparisons test was used for comparisons among multiple groups: $^*P < 0.05$.

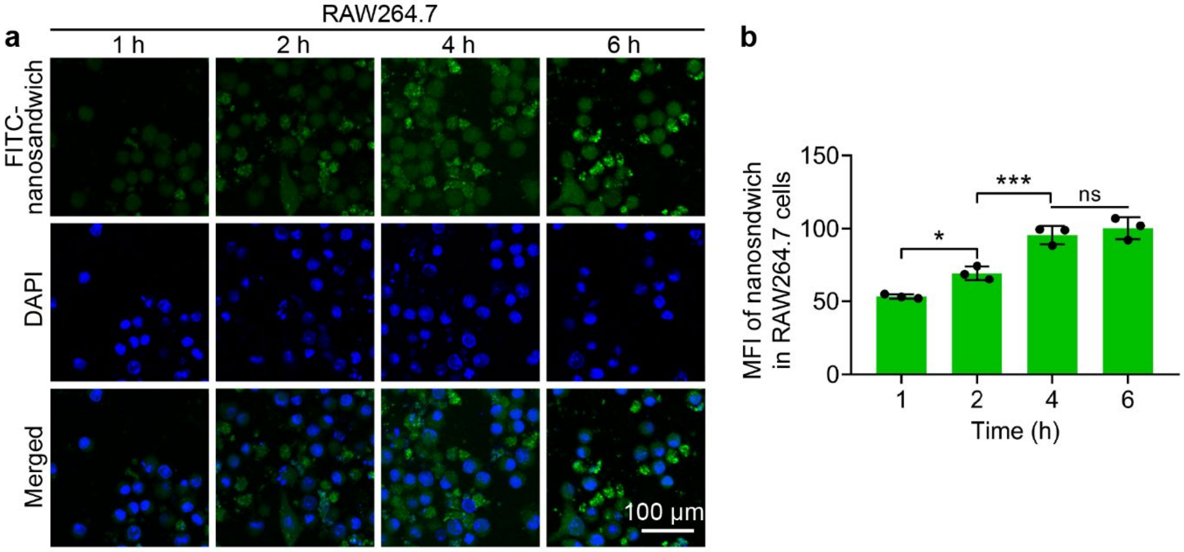

Figure S6. Cellular uptake of P3-HA/PM@BP nanosandwich by RAW264.7 cells after different treatment time. (**a**) CLSM images of RAW264.7 cells treated with 30 μg/mL of FITC-labeled P3-HA/PM@BP nanosandwich (green) for different time, the nuclei were stained with DAPI (blue). Scale bar = 100 μm. (**b**) MFI of internalized nanosandwich in RAW264.7 cells quantified by FCM (n = 3). One-way ANOVA followed by Tukey's multiple comparisons test was used for comparisons among multiple groups: ns indicated $P > 0.05$, *$P < 0.05$, ***$P < 0.001$.

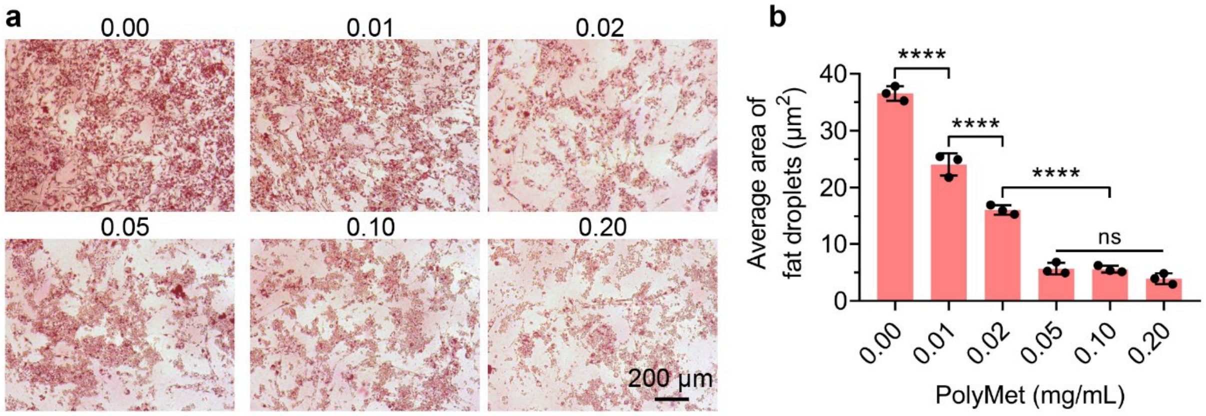

Figure S7. Determination of the treatment concentration of PolyMet on 3T3-L1 cells. (**a**) Oil Red O-stained images of 3T3-L1 after treatments with different concentrations of PolyMet (0.00, 0.01, 0.02, 0.05, 0.10, and 0.20 mg/mL). Scale bar = 200 µm. (**b**) The average area of fat droplets quantified by Image J (*n* = 3). One-way ANOVA followed by Tukey's multiple comparisons test was used for comparisons among multiple groups: ns indicated $P > 0.05$, ****$P < 0.0001$.

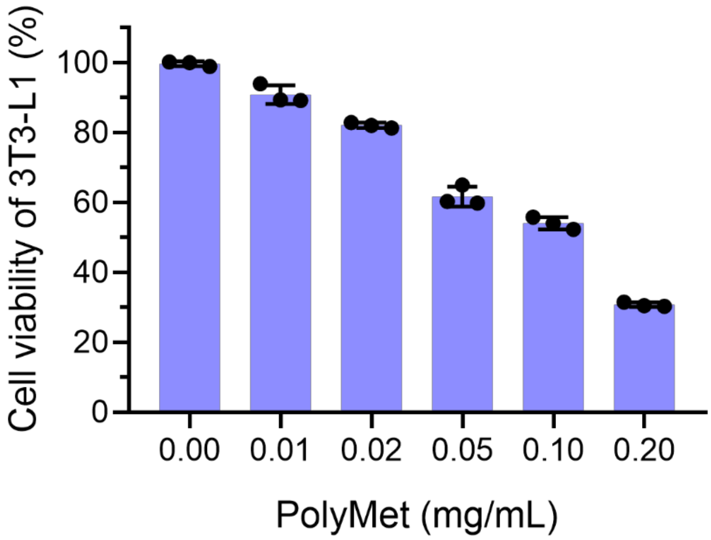

Figure S8. Cytotoxic assay of 3T3-L1 cells treated with various concentrations of PolyMet (0.00, 0.01, 0.02, 0.05, 0.10, and 0.20 mg/mL) for 4 days (*n* = 3).

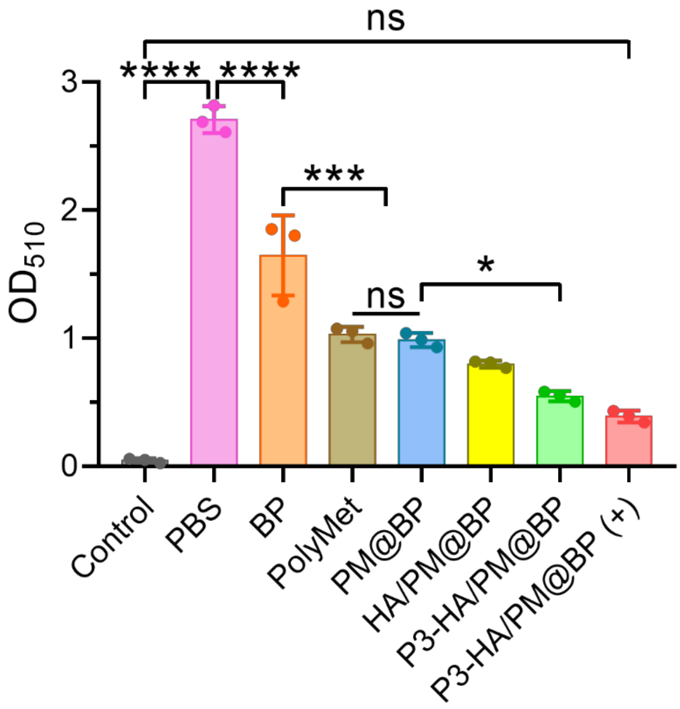

Figure S9. The optical density (OD) of Oil Red O eluted with isopropanol detected at 510 nm after different treatments ($n = 3$). One-way ANOVA followed by Tukey's multiple comparisons test was used for comparisons among multiple groups: ns indicated $P > 0.05$, *$P < 0.05$, ***$P < 0.001$, ****$P < 0.0001$.

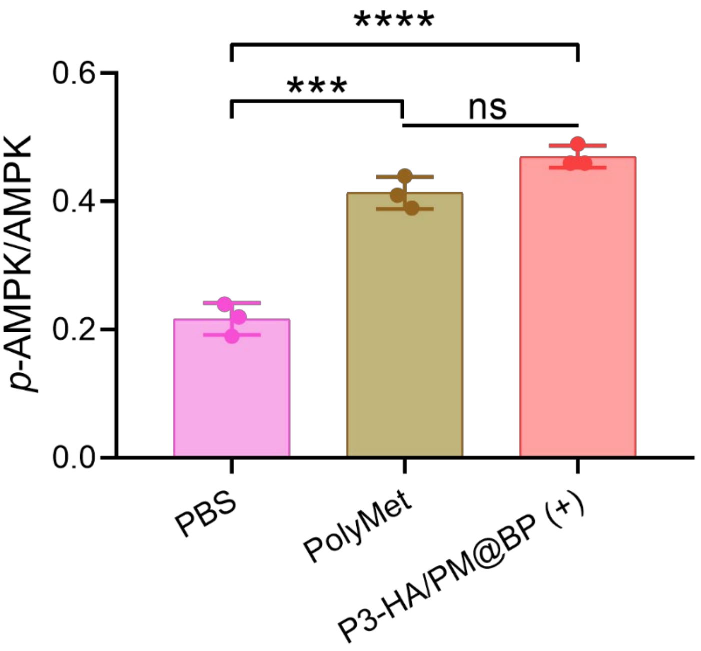

Figure S10. Quantification of the protein expression of phosphorylated AMPK (*p*-AMPK) in 3T3-L1 cells after treatments by Western blotting assay ($n = 3$). One-way ANOVA followed by Tukey's multiple comparisons test was used for comparisons among multiple groups: ns indicated $P > 0.05$, ***$P < 0.001$, ****$P < 0.0001$.

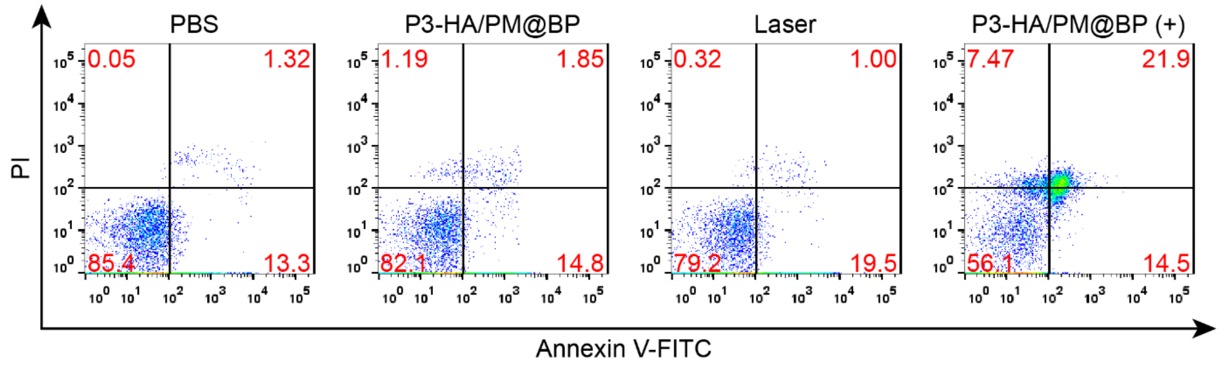

Figure S11. FCM analysis of apoptotic RAW264.7 cells. RAW264.7 cells were respectively treated with PBS, P3-HA/PM@BP nanosandwich, laser (808 nm, 1.5 W/cm^2, 10 min), and P3-HA/PM@BP (+). Cells were double-stained with FITC-Annexin V/PI for FCM analysis.

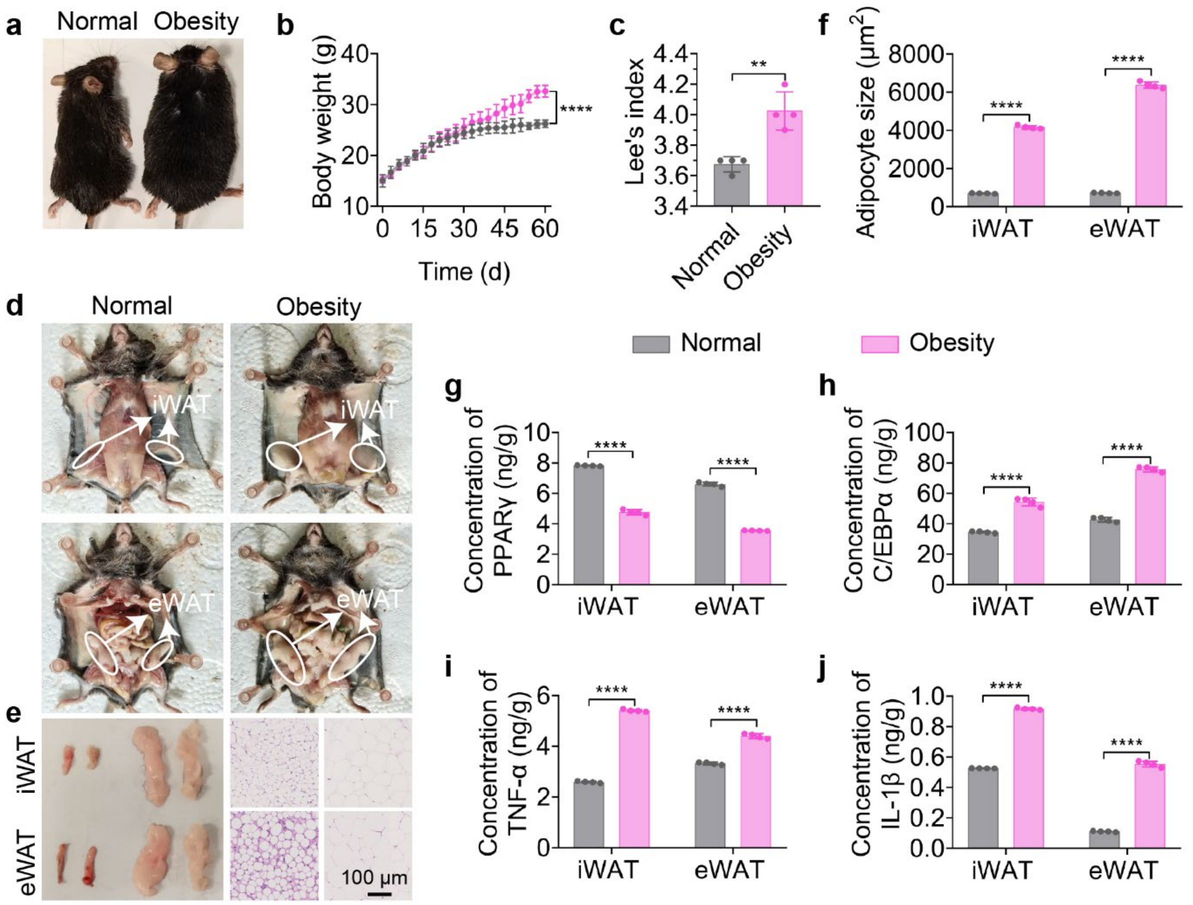

Figure S12. Establishment of DIO mice model and the difference between normal and obese mice after 60 days of diet induction in (**a**) body size, (**b**) body weight, and (**c**) Lee's index (n = 4). The (**d**) anatomical, (**e**) photographs and H&E-stained images of excised iWAT and eWAT in normal and obese mice, and (**f**) the corresponding adipocyte sizes in H&E-stained images evaluated by Image J software (n = 4). Scale bar =100 µm. The concentrations of (**g**) PPARγ, (**h**) C/EBPα, (**i**) TNF-α, and (**j**) IL-1β tested by ELISA (n = 4). Unpaired t test was used for comparisons between two groups: **$P < 0.01$, ****$P < 0.0001$.

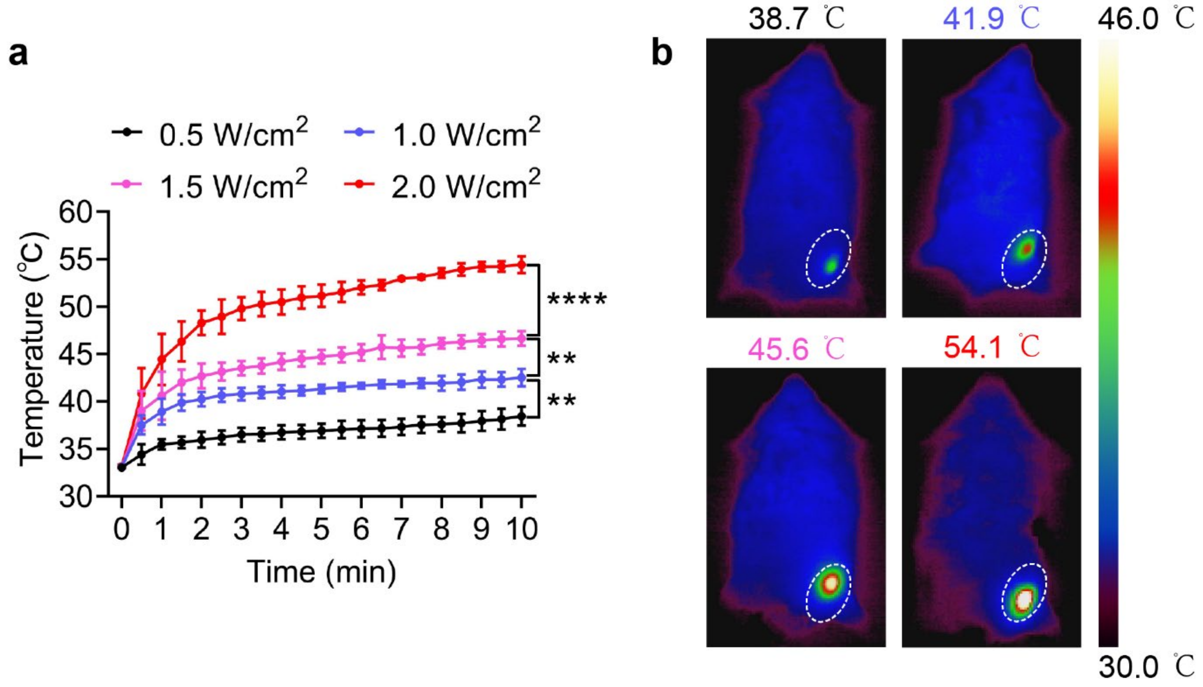

Figure S13. Real-time temperature profiles of iWAT at different laser irradiation powers. (**a**) Temperature changes (n = 3) and (**b**) thermographs of iWAT after NIR irradiation at different powers. One-way ANOVA followed by Tukey's multiple comparisons test was used for comparisons among multiple groups: $^{**}P < 0.01$, $^{****}P < 0.0001$.

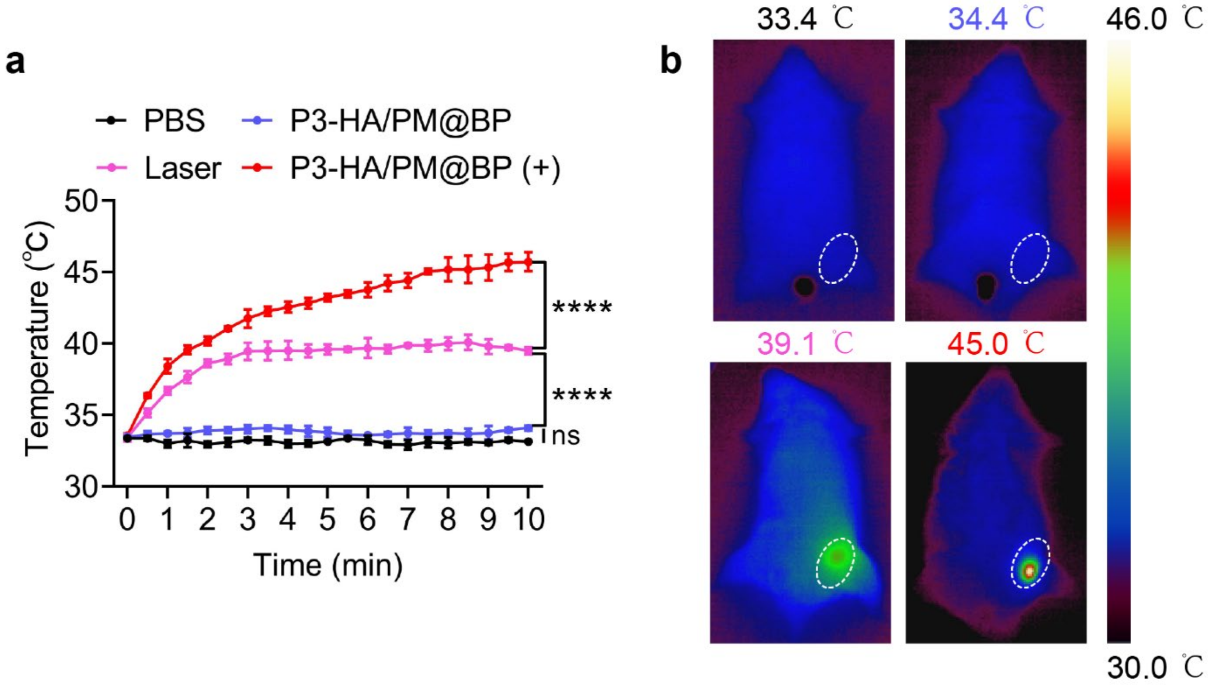

Figure S14. Photothermal effect of P3-HA/PM@BP nanosandwich *in vivo*. (**a**) Temperature changes (n = 3) and (**b**) thermographs of iWAT after different treatments. One-way ANOVA followed by Tukey's multiple comparisons test was used for comparisons among multiple groups: ns indicated $P > 0.05$, ****$P < 0.0001$.

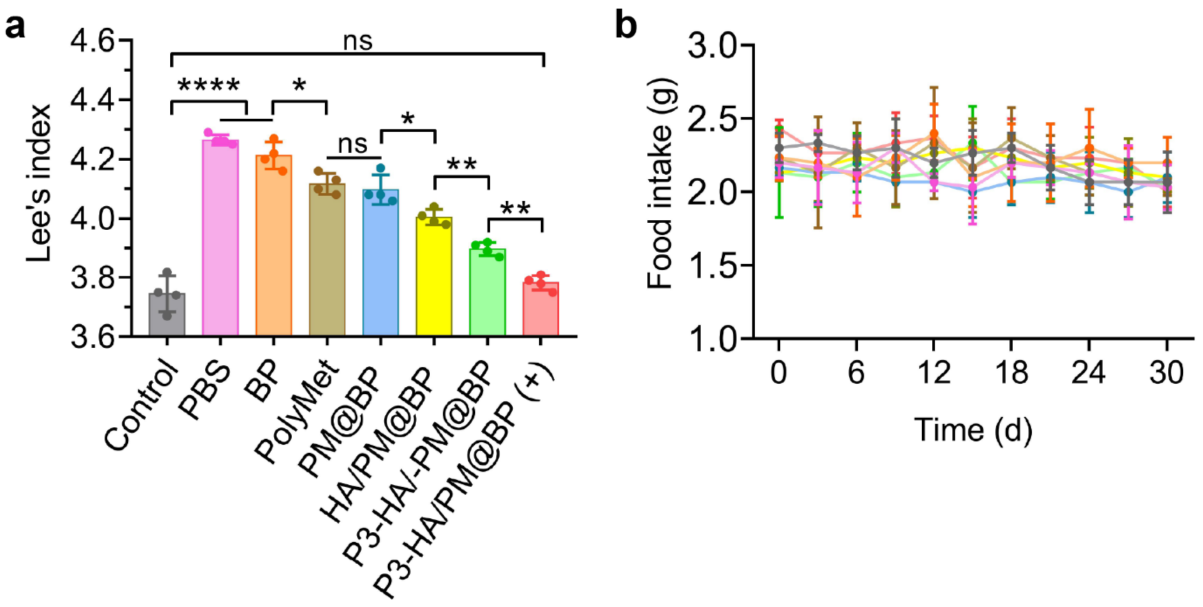

Figure S15. (**a**) Lee's index and (**b**) food intake of DIO mice after different treatments (n = 4). One-way ANOVA followed by Tukey's multiple comparisons test was used for comparisons among multiple groups: ns indicated $P > 0.05$, *$P < 0.05$, **$P < 0.01$, ****$P < 0.0001$.

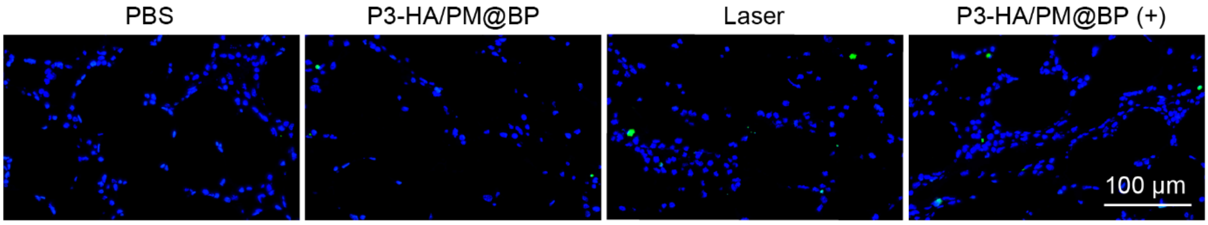

Figure S16. TUNEL staining images of eWAT (green) after different treatments. Scale bar = 100 μm.

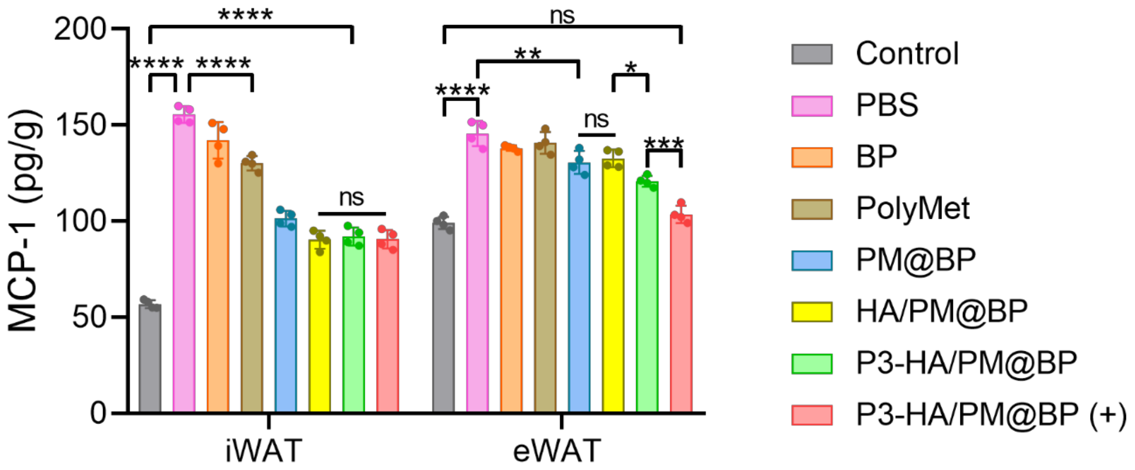

Figure S17. The levels of MCP-1 in iWAT and eWAT after different treatments ($n = 4$). One-way ANOVA followed by Tukey's multiple comparisons test was used for comparisons among multiple groups: ns indicated $P > 0.05$, $^{*}P < 0.05$, $^{**}P < 0.01$, $^{***}P < 0.001$, $^{****}P < 0.0001$.

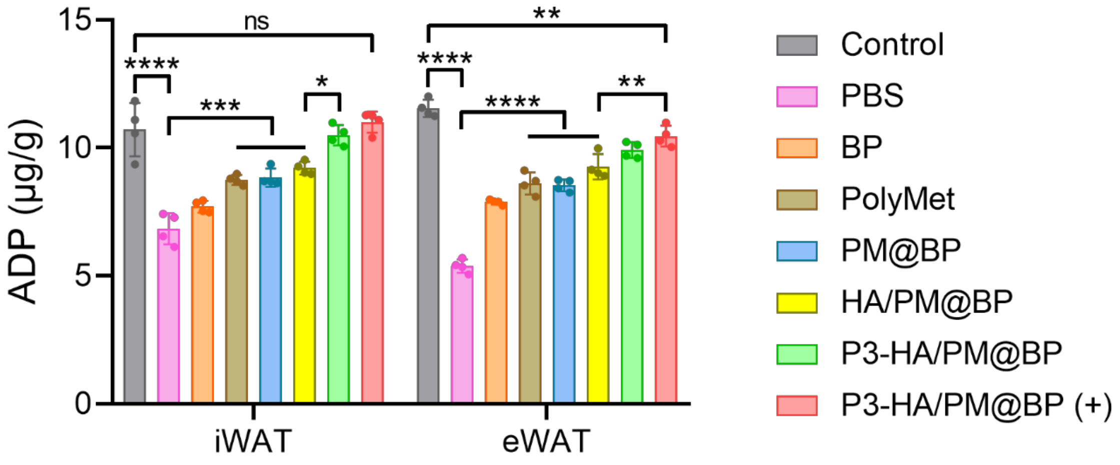

Figure S18. The levels of ADP in iWAT and eWAT after different treatments (n = 4). One-way ANOVA followed by Tukey's multiple comparisons test was used for comparisons among multiple groups: ns indicated $P > 0.05$, *$P < 0.05$, **$P < 0.01$, ***$P < 0.001$, ****$P < 0.0001$.

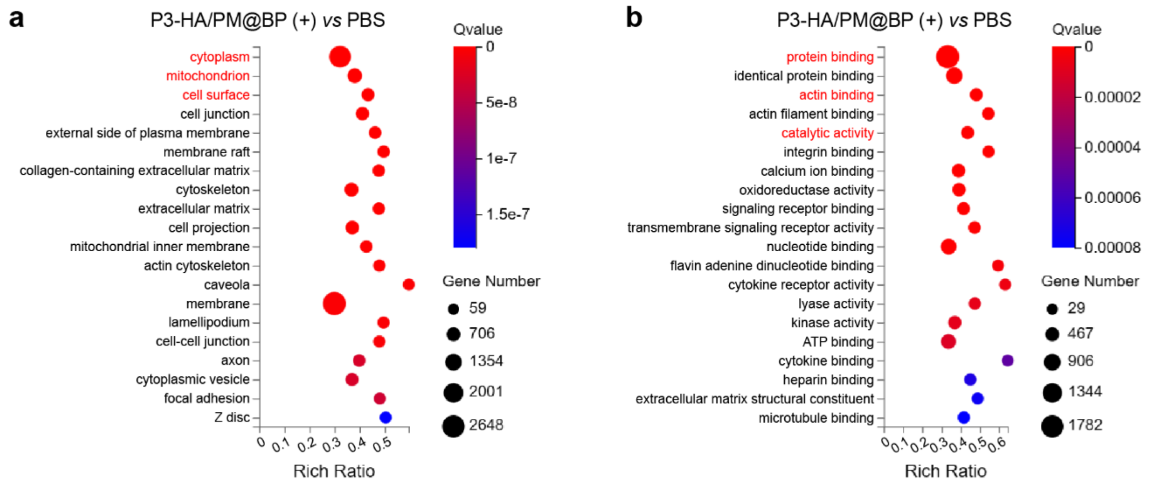

Figure S19. (**a**) The GO_CC and (**b**) GO_MF enrichment analysis of differentially expressed genes (DEGs) in (P3-HA/PM@BP (+) *vs* PBS) group. GO_CC: Gene Ontology_Cell Component; GO_MF: Gene Ontology_Molecular Function.

Table S1. The primary antibodies and secondary antibodies are as follow.

Product	Supplier	Catalogue number	Dilution
**Primary antibody:**			
**Western Blot:**			
Anti-phospho-AMPKα (Thr172) antibody	Cell Signaling Technology	2535	1:1000
Anti-AMPKα1 antibody	Cell Signaling Technology	2795	1:1000
Anti-*β*-actin antibody	Abcam	ab8226	1μg/ml
**Immunohistochemistry：**			
Anti-F4/80 biotin-conjugated antibody	Servicebio	Q61549	1:500
Anti-UCP1 biotin-conjugated antibody	Servicebio	P12242	1:200
**Immunofluorescence：**			
Rabbit Anti-PHB antibody	Proteintech	10787-1-AP	1:200
Rabbit Anti-CD44 antibody	Biocentury	AG1491	1:50
**Flow cytometry:**			
FITC-labeled anti-F4/80 antibody	Elabscience	E-AB-F0995C	1:20
APC-labeled anti-CD206 antibody	Elabscience	E-AB-F1135E	1:20
PE-labeled anti-CD86 antibody	Elabscience	E-AB-F0994UD	1:100
PE-labeled anti-CD206 antibody	Elabscience	E-AB-F1135D	1:20
APC-labeled anti-CD86 antibody	Elabscience	E-AB-F0994UE	1:100
**Secondary antibody：**			
Goat Anti-Rabbit IgG H&L (HRP)	Abcam	ab6721	1:2000
Goat Anti-Mouse IgG H&L (HRP)	Abcam	ab6789	1:2000
Streptavidin-HRP antibody	Servicebio	G3431-1	1:200
Cy3-labeled Goat Anti-Rabbit antibody	Biocentury	P0183	1:100

Table S2. Assay kits used are as follow.

Assay kit	Catalogue number	Supplier
PPARγ ELISA kit	MB-6231A	Biocloon
C/EBPα ELISA kit	MB-6480A	Biocloon
ADP ELISA kit	MB-3283A	Biocloon
MCP-1 ELISA kit	MB-2818A	Biocloon
TNF-α assay kit	MB-2868A	Biocloon
IL-1β assay kit	MB-2776A	Biocloon
IL-6 assay kit	MB-2899A	Biocloon
IL-10 assay kit	MB-2912A	Biocloon
TG assay kit	A110-1-1	Nanjing Jiancheng
TC assay kit	A111-1-1	Nanjing Jiancheng
HDL-C assay kit	A112-1-1	Nanjing Jiancheng
LDL-C assay kit	A113-1-1	Nanjing Jiancheng
FFA assay kit	A042-1-1	Nanjing Jiancheng
AST assay kit	C010-2-1	Nanjing Jiancheng
ALT assay kit	C009-2-1	Nanjing Jiancheng
BUN assay kit	C013-1-1	Nanjing Jiancheng
CRE assay kit	C011-2-1	Nanjing Jiancheng
UA assay kit	C012-2-1	Nanjing Jiancheng
Annexin V-FITC/PI cell apoptosis detection kit	E-CK-A211	Elabscience

Reference

[1] Y. Mei, L. Tang, L. Zhang, J. Hu, Z. Zhang, S. He, J. Zang, W. Wang, *Mater. Today* **2022**, *60*, 52.
